# Excited‐State Structural Dynamics of the Cubane‐Type Metal Cluster [Cu_4_I_4_(py)_4_] Explored by Time‐Resolved X‐Ray Liquidography

**DOI:** 10.1002/advs.202414970

**Published:** 2025-02-14

**Authors:** Doyeong Kim, Hosung Ki, Donghwan Im, Yunbeom Lee, Seonggon Lee, Jun Heo, Seunghwan Eom, Eun Hyuk Choi, Doo‐Sik Ahn, Hyotcherl Ihee

**Affiliations:** ^1^ Department of Chemistry Korea Advanced Institute of Science and Technology (KAIST) Daejeon 34141 Republic of Korea; ^2^ Center for Advanced Reaction Dynamics Institute for Basic Science (IBS) Daejeon 34141 Republic of Korea

**Keywords:** cubane‐type metal cluster, molecular structural dynamics, reaction dynamics, time‐resolved X‐ray liquidography, time‐resolved X‐ray solution scattering

## Abstract

Cubane‐type metal clusters respond uniquely to stimuli like light and electric potential, resulting in behaviors such as crystal‐to‐crystal phase transitions. While structural adaptability is known to be linked to these responses, direct experimental evidence for the associated structural changes has been missing. This study addresses this gap by examining the structural dynamics of the copper(I) iodide cubane (Cu_4_I_4_(py)_4_, py = pyridine) upon photoexcitation using time‐resolved X‐ray liquidography. The results reveal: 1) 100 picoseconds (ps) after excitation, two distinct excited states—the cluster‐centered triplet (^3^CC) state and the (metal+halide)‐to‐ligand charge transfer triplet (^3^(M/X)LCT) state—are present; 2) the ^3^(M/X)LCT state decays with an apparent time constant of 1.21 ns, primarily transitioning to the ^3^CC state, with a small fraction undergoing decay to the ground state (GS); and 3) the ^3^CC state eventually returns to the GS. The molecular structures, provided for these states serve as benchmarks for theoretical studies. Importantly, the ^3^CC structure exhibits significant distortion in the Cu_4_I_4_ core and reduced symmetry, findings that are unanticipated by previous models. This comprehensive investigation deepens the understanding of the structural transformations occurring upon photoexcitation, with a potential impact on future applications of these compounds as versatile components in photosensitive metal–organic frameworks.

## Graphical abstract

The structural dynamics of cubane‐type metal clusters are monitored using time‐resolved X‐ray solution scattering, revealing their structural adaptability under photoexcitation. Notably, a large structural distortion accompanied by symmetry lowering—distinct from the previously reported result—is observed, along with a transition between two emissive states exhibiting highly distinct structural differences.

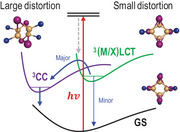

## 1 Introduction

**Figure 1:**
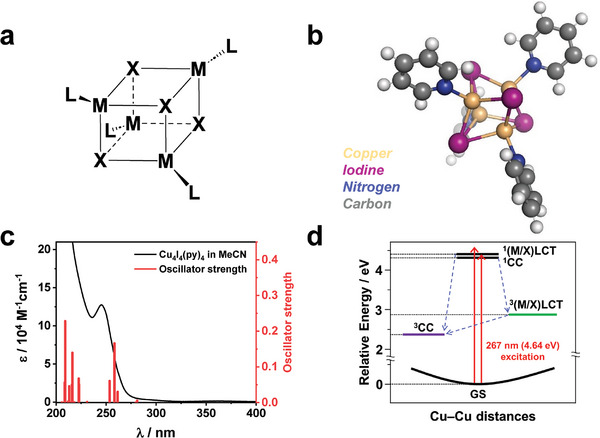
Molecular structure and absorption spectrum of Cu_4_I_4_(py)_4_. a) Simplified structure of a cubane‐type metal halide cluster. Here, the symbols M, X, and L refer to metal, halide, and ligand, respectively. b) Structure of Cu_4_I_4_(py)_4_, with color codes: I (purple), Cu (brown), N (blue), C (gray), and H (white). c) Molar extinction coefficient and calculated oscillator strength of Cu_4_I_4_(py)_4_ in acetonitrile solution. The measured molar extinction coefficient is represented by the black curve, while the oscillator strengths for singlet‐singlet transitions, calculated using TD‐DFT, are depicted as red bars. d) Schematic diagram illustrating the initial state reached through 267 nm excitation and the excited states targeted for observation in this experiment.

Cubane‐type metal cluster compounds have attracted significant attention for their unique responses to external factors such as light, solvent, pressure, and electric potential.^[^ ^1^, ^2^, ^3^ ^]^ These stimuli induce various distinct behaviors, including magnetic phase transitions and crystal‐to‐crystal transformations.^[^ ^4^, ^5^ ^]^ For this reason, cubane‐type complexes have found applications in functional materials, where they serve as secondary building units in metal–organic frameworks and as catalysts. Notable examples of these complexes include metal‐oxides (M_4_O_4_) and metal‐halides (M_4_X_4_), with a representative structure shown in **Figure** **1**a.^[^ ^5^, ^6^, ^7^, ^8^, ^9^ ^]^

A representative model system for cubane‐type metal clusters is Cu_4_I_4_(py)_4_, a copper(I) iodide tetramer with pyridine ligands. This complex has been the subject of comprehensive theoretical and spectroscopic investigations,^[^ ^1^, ^10^, ^11^, ^12^, ^13^, ^14^ ^]^ primarily due to its dual luminescence.^[^ ^15^ ^]^ The molecular structure of the cluster is shown in Figure 1b. From the spectroscopic studies, it was reported that the complex shows dual emission, which was assigned to ^3^CC state and ^3^(M/X)LCT state.^[^ ^1^, ^12^, ^13^, ^14^ ^]^ The low‐energy emission band at ≈550–650 nm is dominant at room temperature, attributed to ^3^CC emission consisting of a mixture of halide‐to‐metal charge transfer (^3^XMCT) and metal cluster‐centered (d^10^ Cu → d^9^s^1^ Cu) characteristics.^[^ ^12^ ^]^ The high‐energy emission band in the blue region (400–450 nm) dominates at 77 K and is assigned to the state possessing a mixed feature of both halide‐to‐ligand charge transfer (^3^XLCT) and metal‐to‐ligand charge transfer (^3^MLCT) transitions.^[^ ^1^ ^]^ Here, we term the state of the latter, a mixed state of ^3^XLCT and ^3^MLCT, as ^3^(M/X)LCT (See the “Characterization of the two emissive states in Cu_4_I_4_(py)_4”_ section in Supporting Information.

The physical and chemical characteristics of cubane‐type metal clusters primarily arise from their structures, particularly the metal–metal distances within the cubane framework.^[^ ^12^, ^16^ ^]^ Therefore, it becomes essential to monitor and investigate the structural changes within the cubane framework associated with the dual luminescence dynamics, aiming to unravel the underlying mechanism behind this phenomenon. Until now, the study of the emissive state's structure of Cu_4_I_4_(py)_4_ has solely relied on theoretical investigations, lacking direct experimental evidence. Recognizing this gap, we employed time‐resolved X‐ray liquidography (TRXL),^[^ ^17^, ^18^, ^19^, ^20^, ^21^ ^]^ also known as time‐resolved X‐ray solution scattering, to investigate Cu_4_I_4_(py)_4_ in acetonitrile solution. TRXL is a powerful technique capable of directly capturing the structural changes of molecules in solution.^[^ ^22^, ^23^, ^24^, ^25^, ^26^ ^]^ Importantly, the TRXL signal displays exceptional sensitivity to the distances between heavy atoms, such as Cu and I, rendering it an optimal method for capturing structural changes within the Cu_4_I_4_ frame in Cu_4_I_4_(py)_4_. Our TRXL experiments on the copper‐iodide cubane cluster unveiled three key findings: i) Upon photoexcitation, the two excited states, characterized by ^3^CC and ^3^(M/X)LCT features, are rapidly reached within the first 100 ps; ii) Through internal conversion, the ^3^(M/X)LCT transitions into ^3^CC state, followed by a recovery to the GS; iii) The ^3^CC state, as determined through experiment, exhibits a more pronounced structural change, including a reduction in symmetry, diverging from the results reported in a theoretical study.^[^ ^14^ ^]^

## 2 Results and Discussion

### 2.1 Absorption Spectrum of Cu_4_I_4_(py)_4_ and Assignment of Transitions Associated with 267 nm Excitation

To investigate the photophysical properties of Cu_4_I_4_(py)_4_, we conducted density functional theory (DFT) and time‐dependent density functional theory (TD‐DFT) calculations. The corresponding molecular diagram of ground state Cu_4_I_4_(py)_4_ is illustrated in Figure S1 (Supporting Information). Briefly, the HOMO to HOMO‐4 share similar characteristics, featuring a mix of the 4p orbital of I and the 3d orbital of Cu. LUMO to LUMO+3 predominantly exhibit π orbital character localized in the py ligands. As a result, the previously mentioned (M/X)LCT transition likely corresponds to an electronic transition from the HOMO‐to‐HOMO‐4 to the LUMO‐to‐LUMO+3. In contrast, the LUMO+4 consists of a mixture of the 5s orbital of Cu and the 4p orbital of I. Note that the LUMO+4 shows bonding character among the four Cu atoms and antibonding character between all Cu and I atoms. Considering the bonding nature of the LUMO+4 between Cu atoms, the transition between HOMO‐to‐HOMO‐4 and the LUMO+4 is expected to exhibit metal cluster‐centered characteristics. Figure S2 (Supporting Information) presents the natural transition orbitals (NTOs) corresponding to the two bright transitions.

The calculated oscillator strengths to the singlet excited states, along with the measured absorption spectrum of Cu_4_I_4_(py)_4_, are shown in Figure 1c. Notably, a prominent absorption peak is observed at ≈247 nm. Our TD‐DFT calculations reveal energy bands with significant oscillator strengths (> 0.05) in the range of 253 to 258 nm, as detailed in Table S1 (Supporting Information). The calculated transition energies exhibit a slight red‐shift compared to the measured absorption spectra. Considering a typical deviation of 0.2–0.4 eV between excitation energies from TD‐DFT^[^ ^27^ ^]^ and experimental values, the calculated energies align well with the absorption spectrum. To gain insight into the character of the electronic transitions assigned via TD‐DFT calculations, we performed transition density matrix^[^ ^28^, ^29^ ^]^ analysis for the five significant transitions near the prominent absorption peak. The results, presented in Figures S3–S4 and Tables S2–S3 (Supporting Information), suggest a complex nature of this absorption band, with various types of transitions contributing in a mixed manner. The transitions contributing to the absorption band can be classified into the following two categories, reflecting the primary character of the transition: i) Transitions with CC character, where CC properties account for more than 80% of the total characteristics (S_7_ to S_9_), and ii) Transitions primarily characterized by (M/X)LCT transitions, accounting for more than 50% of the total characteristics (S_12_ to S_13_). A detailed description of the characteristics of each type of transition is provided in the “Characterization of the two emissive states in Cu_4_I_4_(py)_4”_ section in Supporting Information.

We used an excitation wavelength of 267 nm, corresponding to the shoulder of the lowest electronic absorption band, as confirmed by the measured absorption spectrum and TD‐DFT calculations. Given that these bands possess high oscillator strengths and their energies significantly overlap, we expected that both transitions—those with CC character and those with (M/X)LCT character—would occur. Figure 1d depicts a schematic illustration of the energy states accessible through the 267 nm excitation and the states we aim to investigate in the TRXL experiment. We note that limitations in the instrument response function (IRF) in this TRXL experiment, which is ≈100 ps, did not allow capturing the initial moments of the reaction. This includes the expected dynamics that start with an initial population of cluster‐centered singlet (^1^CC) or (metal+halide)‐to‐ligand charge transfer singlet (^1^(M/X)LCT) states, followed by a transition to emissive triplet states.

### 2.2 TRXL Data and SVD Analysis

To track the structure of the two emissive states and their subsequent dynamics, we conducted a TRXL experiment on Cu_4_I_4_(py)_4_ in acetonitrile solution. The TRXL scattering curves are influenced by three types of structural changes: changes in the solute, changes in the interatomic distances between the solute molecule and its surrounding solvent molecules (cage), and structural changes in the bulk solvent. Given our focus on the structural changes related to the solute molecules and cages, we applied the projection to extract the perpendicular component (PEPC) method. This technique effectively screens out the influence of bulk solvent, leaving only the solute and cage contributions, by projecting the data into a subspace orthogonal to the solvent terms, as detailed in the previous work.^[^ ^30^ ^]^ The details regarding the subtraction process of the solvent term using the PEPC method are described in the “Projection to extract the perpendicular component method” section in SI. In addition to the solvent heating component, other factors—such as laser fluence‐dependent artifacts—may have contributed to the data, complicating the extraction of solute‐related kinetics from the TRXL data. To address this issue, we performed additional TRXL experiments, measuring solvent responses at two different laser fluences. This approach allowed us to identify and isolate an artifact that could not be accounted for by the two known heating components. Further details on the methodology used to extract and correct for this additional artifact are provided in the “Data processing” section of Supporting Information. The profiles of the solvent heating components, along with the high‐fluence‐induced artifact, are presented in Figure S5 (Supporting Information).

**Figure 2:**
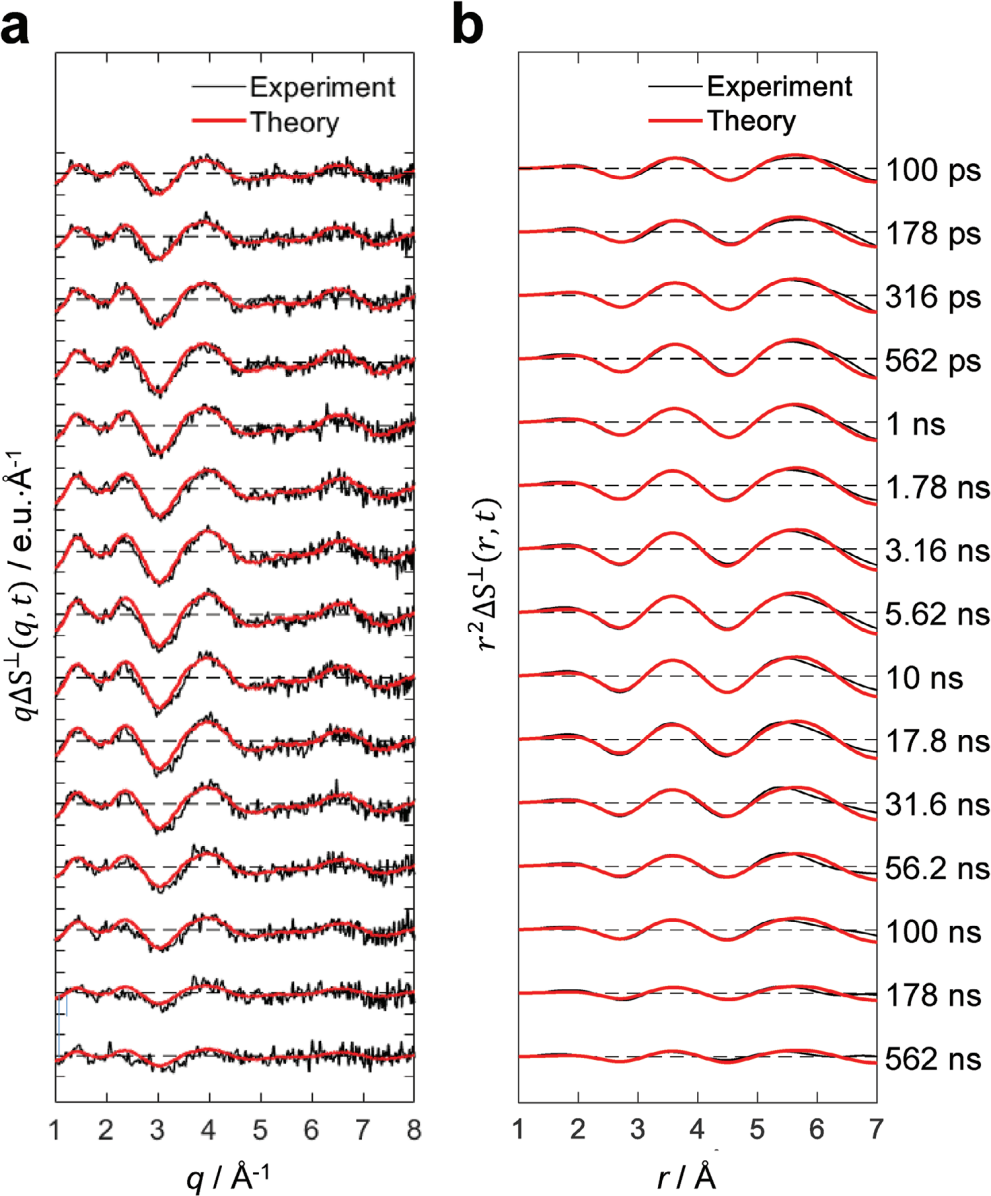
Time‐resolved solvent‐contribution‐free difference X‐ray scattering curves of Cu_4_I_4_(py)_4_ in acetonitrile. a) Experimental difference scattering curves (black) are plotted together with simulated theoretical fits (red). The theoretical fits were obtained using linear combination fitting (LCF) of ΔS^⟂^(*q*, *t*) at each time delay. b) Difference radial distribution function, *r* ^2^ΔS^⟂^(*r*, *t*), obtained by Fourier sine transformation of *q*ΔS^⟂^(*q*, *t*) in (a).

The resulting PEPC‐treated data, which we refer to as a solvent‐contribution‐free signal, ΔS^⟂^(*q*, *t*), are displayed in **Figure** **2**. ΔS^⟂^(*q*, *t*) is a function of time delay (*t*) and the magnitude of the momentum transfer vector (*q*), represented by *q* = (4π/*λ*)sin(2*θ*/2). Here, *λ* denotes the X‐ray wavelength, and 2*θ* signifies the scattering angle. The raw data, ΔS(*q*, *t*), before removing the solvent contribution using this method, are also illustrated in Figure S6 (Supporting Information). The theory curves in Figure 2 and Figure S6 (Supporting Information) were generated using a linear combination fit of the solute term, cage term, and solvent term, based on the optimized molecular structure obtained through structure refinement. To highlight the region where the structural changes of the solute molecules primarily contribute, namely the high‐*q* region, we multiplied ΔS^⟂^(*q*, *t*) by *q* to generate *q*ΔS^⟂^(*q*, *t*). A clear oscillatory pattern emerges in the high‐*q* region within a time delay of 100 ps. The rapid signal development signifies the population of excited states with structures distinct from the GS, occurring within the IRF of the experiment.

To gain further insights from ΔS^⟂^(*q*, *t*), we applied singular value decomposition (SVD) analysis on ΔS^⟂^(*q*, *t*). SVD decomposes the original signal matrix into a product of the left singular vectors (LSVs), singular values, and right singular vectors (RSVs). The resulting LSVs depict the time‐independent components in *q*‐space that comprise ΔS^⟂^(*q*, *t*), the RSVs are the temporal changes of both shape and amplitude of the signal, thereby representing the kinetics, and singular values indicate the relative contributions of the corresponding components. More detailed information regarding SVD analysis can be found in the “Singular value decomposition (SVD) analysis” section in Supporting Information. The obtained RSVs, LSVs, and singular values are presented in Figure S7a–c (Supporting Information). The SVD analysis revealed two major signal components, suggesting the involvement of two distinct species in the reaction pathways. To extract the kinetics of these two species, we performed a global fit of the first two major RSVs using a sum of exponential functions, each incorporating a constant term, with the same time constants applied across both RSVs. For the fitting of the RSV1, the constant term was fixed at zero. Analysis with varying numbers of exponential functions indicated that a sum of two exponential functions satisfactorily fits the two RSVs, as depicted in Figure S8 (Supporting Information). The exponential fitting, shown in Figure S7d (Supporting Information), determined two time constants: 1.21 ± 0.61 ns and 202 ± 37 ns.

### 2.3 Elucidating Reaction Pathways: Structural Analysis and Kinetic Modeling

**Figure 3:**
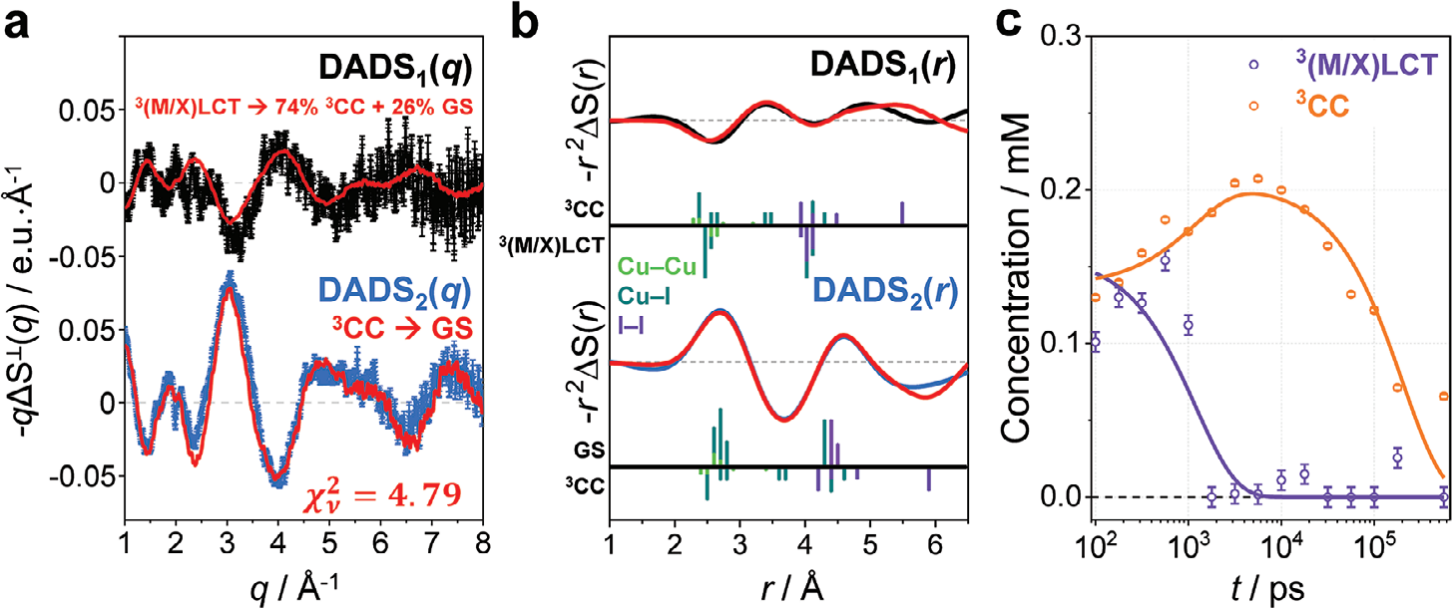
Comprehensive analysis of decay‐associated difference scattering curves (DADSs) for Cu_4_I_4_(py)_4_. a) Experimental and theoretical solvent‐contribution‐free DADSs in *q*‐space. The experimental DADS_1_(*q*) and DADS_2_(*q*) are shown in black and blue, respectively. The theoretical DADSs, obtained through structure refinement and depicted in red, are overlaid on the experimental DADSs for comparison. The y‐axis is expressed in electron unit per molecule. b) *r* ^2^ΔS(*r*)s obtained through Fourier sine transforms of *q*DADS_1_(*q*) and *q*DADS_2_(*q*), illustrating structural changes in *r*‐space. Each panel displays interatomic distances within the Cu_4_I_4_ framework as vertical bars, distinguishing positive (above) from negative (below) contributions to the signals. The bars are color‐coded by atom type and their heights are scaled according to atomic numbers to accurately reflect the contribution of the corresponding atomic pair to the scattering signal. The corresponding structures for these distances are labeled on the left side of the figure. For DADS_1_(*r*), only the changes associated with the major transition were represented using bars. c) Time‐dependent concentration profiles of molecules occupying each assigned excited state (e.g., ^3^(M/X)LCT and ^3^CC states). Solid lines represent biexponential fits using time constants derived from kinetic analysis of RSVs obtained through SVD analysis of ΔS^⟂^(*q*, *t*).

Subsequently, we extracted and analyzed the decay‐associated difference scattering curves (DADS(*q*)) to investigate the dynamic origins of the two time constants. The term, DADS, is named after “decay associated spectra”, which is commonly used in the field of spectroscopy to represent a spectrum corresponding to a specific decay time constant. The details about the DADS analysis is described in the “Kinetic analysis” section in SI. Each *i*‐th DADS (DADS*_i_* (*q*)) stands for the difference X‐ray scattering curve of the solution before and after the dynamics occurring with the corresponding *i*‐th time constant, τ*_i_*. The resulting DADS(*q*)s multiplied by *q* are described in **Figure** **3**a and Figure S9 (Supporting Information).

Considering that a constant term was included during the exponential fitting analysis of RSVs, we also performed an analysis using three DADSs, including DADS_3_, which corresponds to a long‐lived species. The result of this analysis, shown in Figure S10 (Supporting Information), revealed that DADS_3_ contains only negligible structural signals, indicating that all intermediates decay to the ground state at least within a time constant of 202 ns. Related discussions are provided in the Supporting Information section titled “Investigation of the potential for long‐lived photochemical products that do not decay within the observed time window”.

To identify the structural changes corresponding to the obtained DADS*_i_* (*q*), we first generated candidate structures that are potentially involved in the photoreaction pathways. Subsequently, we calculated theoretical difference scattering curves corresponding to various transitions between these candidate structures. By comparing the candidate theoretical difference scattering curves with the experimentally obtained DADS*_i_* (*q*), we identified the structural transition that best matches the observed DADS*_i_* (*q*). The comparisons are shown in Figure S11 (Supporting Information). We considered two candidate structures, corresponding to the ^3^CC and ^3^(M/X)LCT excited states, respectively. The structures of the two excited states, as well as the ground state (GS), were obtained using DFT calculations. At this stage, we calculated the theoretical difference scattering curves using the DFT‐optimized structures without further refinement or adjustment of structural parameters to fit the experimental data (In a later stage, the structural parameters are also refined via structure refinement). The molecular orbitals and structural parameters of the DFT‐optimized candidate structures can be found in Figure S1 and Table S4 (Supporting Information). Additional information regarding the DFT calculations is available in the “Density functional theory (DFT) and time‐dependent density functional theory (TD‐DFT) calculation” section in SI. Upon comparing the candidate theoretical curves with the DADSs (Figure S11, Supporting Information), we assigned DADS_1_(*q*), which corresponds to the 1.21 ns time constant, to the transition from ^3^(M/X)LCT to ^3^CC, and DADS_2_(*q*), with the 202 ns time constant, to the transition from ^3^CC to GS, respectively.

It is worth noting that previous studies^[^ ^1^, ^31^ ^]^ proposed a model, based on their observation of the concentration‐dependent high‐energy emission rate from the ^3^(M/X)LCT state, that consists of two competing pathways: 1) emissive decay directly to the ground state (^3^(M/X)LCT → GS), and 2) a bimolecular energy transfer process from one Cu_4_I_4_(py)_4_ molecule in the ^3^(M/X)LCT state to another in the GS, producing the ^3^CC state (^3^(M/X)LCT + GS → GS + ^3^CC). At higher concentrations, the bimolecular energy transfer pathway dominates, while at lower concentrations, direct decay to the GS prevails. Given that our experimental concentration (5 mm) exceeds even the highest concentration used in prior studies (1.35 mm), it is reasonable to infer that bimolecular energy transfer is the predominant pathway under our experimental conditions.

Noting that the previous measurements^[^ ^1^, ^31^ ^]^ were conducted for Cu_4_I_4_(py)_4_ dissolved in a different solvent, benzene, we performed time‐correlated single‐photon counting (TCSPC) measurements in acetonitrile to investigate whether the two proposed pathways for the decay of ^3^(M/X)LCT state are also operative in acetonitrile. The TCSPC results are presented in Figure S12 and Table S5 (Supporting Information). For these measurements, the samples were excited at 280 nm, corresponding to the same absorption band as the 267 nm wavelength used in our TRXL experiments. The luminescence lifetime was measured at an emission wavelength of 315 nm, which corresponds to the high‐energy emission band among the dual‐emissive bands of Cu_4_I_4_(py)_4_ in acetonitrile at 293 K (see Figure S13, Supporting Information).

The TCSPC results confirmed that both pathways contribute to the decay of the ^3^(M/X)LCT state in acetonitrile. The luminescence lifetime of the ^3^(M/X)LCT state decreased with increasing Cu_4_I_4_(py)_4_ concentration, indicating a concentration‐dependent bimolecular process. This aligns with the bimolecular energy transfer pathway proposed in the previous studies.^[^ ^1^, ^31^ ^]^ Additionally, the decay rate did not converge to zero at near‐zero concentrations, suggesting the presence of a concentration‐independent pathway. Comparison of the TCSPC data collected under ambient air and inert conditions revealed two distinct concentration‐independent processes: 1) oxygen quenching of the triplet excited state, and 2) radiative decay independent of oxygen. These findings corroborate the model proposed in the previous study and provide additional insights into the interplay of concentration‐independent and concentration‐dependent processes in the decay of the ^3^(M/X)LCT state. The details of the TCSPC measurements and the resulting kinetic model are provided in the Supporting Information section, “Understanding the variation in luminescence lifetimes of Cu_4_I_4_(py)_4_: Solvent effects and concentration.”.

The TCSPC measurements further confirmed contributions from two competing pathways: 1) ^3^(M/X)LCT → ^3^CC, and 2) ^3^(M/X)LCT → GS, with quantitative contributions of 74% and 26%, respectively. Based on these results, we established a kinetic model, illustrated in Figure S12d (Supporting Information).

To gain a more intuitive insight into structural changes, we applied Fourier sine transform to DADS*_i_* (*q*)s. A comprehensive description of the procedure for obtaining the difference radial distribution functions, represented as DADS(*r*), is provided in the “Fourier sine transform for converting *q*‐space data into *r*‐space information” section of SI. The resulting DADS*_i_* (*r*)s directly visualize the changes in interatomic distances in real space, as presented in Figure 3b. Here, we inverted the signs of DADS*_i_* (*q*) and DADS*_i_* (*r*) in Figure 3a,b, presenting them as ‐*q*ΔS^⟂^(*q*) and a ‐*r^2^* ΔS*_i_* (*r*) graphs, respectively, to illustrate the contributions of reactants as negative and products as positive. DADS_1_(*q*) reflects a major contribution from the transition from ^3^(M/X)LCT to ^3^CC, as well as a minor contribution from the transition from ^3^(M/X)LCT to GS. Consequently, its reciprocal representation, DADS_1_(*r*), is a superposition of negative contributions from ^3^(M/X)LCT and positive contributions from ^3^CC and GS. The DADS_1_(*r*) exhibits two prominent negative peaks at ≈2.6 and 4.3 Å, attributed to the Cu–Cu and Cu–I distances of the ^3^(M/X)LCT structure, respectively. The long Cu–I distances in the ^3^(M/X)LCT structure contrast with the shorter ones in the ^3^CC structure, which are ≈2.5 Å. The corresponding positive peak of ^3^CC in DADS_2_(*r*) is barely distinguishable, because it is overshadowed by the strong negative contributions from the Cu–Cu and Cu–I distances in the ^3^(M/X)LCT structure. These interatomic distances contributing negatively to the signal, associated with the ^3^(M/X)LCT structure, are present in the vicinity of the interatomic distances contributing positively, associated with the ^3^CC structure. It is worth noting that the ^3^CC structure exhibits a wide distribution of I–I distances ranging from 4.2 to 5.9 Å. Among these distances, the long I–I distances, particularly those around 5.9 Å, make a significant contribution to the pronounced positive peak observed in the high‐*r* region (*r* > 5 Å) of DADS_1_(*r*).

The DADS_2_(*r*) exhibits a clear reversal in sign in comparison to that of DADS_1_(*r*). In other words, positions where DADS_1_(*r*) displayed negative signals exhibit positive signals in DADS_2_(*r*), while positions that exhibit positive signals in DADS_1_(*r*) show negative signals in DADS_2_(*r*). This reversal can be primarily attributed to two main factors: 1) Unlike DADS_1_(*r*), which illustrates the population increase of the ^3^CC state associated with the depletion of the ^3^(M/X)LCT state, DADS_2_(*r*) corresponds to the decay of the ^3^CC state to the GS; 2) The structural similarity between the ^3^(M/X)LCT and GS structure further substantiates this sign reversal. Upon analyzing the distribution of interatomic distances between Cu and I atoms in the ^3^(M/X)LCT and GS structures in Figure 3b, it becomes apparent that these two states exhibit structural similarity. Two prominent positive peaks are observed in DADS_2_(*r*), approximately at 2.7 and 4.5 Å. These peaks represent Cu–Cu (2.6 Å), Cu–I (2.7–2.9 Å), and I–I (4.5 Å) distances in the GS, indicating recovery of GS from ^3^CC.

To extract detailed structural information for each species, we refined the DFT‐optimized structures of the GS, ^3^(M/X)LCT, and ^3^CC states by adjusting specific structural parameters. This refinement aimed to minimize the discrepancy between the theoretical DADS(*q*)s, calculated from the structures of the species, and the experimental DADS(*q*)s.

To identify the most appropriate approach for our structural analysis, we compared the results obtained from three distinct methods: 1) utilizing the DFT‐optimized structures without further refinement, 2) refining both the copper and iodine atom positions, and 3) refining only the iodine atom positions while keeping the copper atoms fixed according to the DFT‐optimized structures. A comparison of the results is illustrated in Figure S14 (Supporting Information), and the structural parameters obtained from each approach are provided in Table S4 (Supporting Information). As shown in Figure S14 (Supporting Information), the third approach—where only the iodine atom positions were refined while the copper positions remained fixed—significantly improved the fitting quality compared to the direct use of the DFT‐optimized structures. Although the second approach, which involved optimizing both copper and iodine positions, provided slightly better fitting quality, it led to the generation of abnormally short Cu–Cu bond distances. In contrast, the third approach avoids this artifact, offering a more chemically reasonable representation of the structural parameters. To balance fitting quality and physical plausibility, we adopted the third approach, fixing the positions of the Cu atoms based on the DFT‐optimized structures while allowing only the positions of the iodine atoms to vary during the refinement process. Further details on the structure refinement process are described in the “Structure refinement by DADS fitting” section in Supporting Information. The resulting structural parameters are provided in Table S4 (Supporting Information). We note that the occurrence of longer metal‐metal distances in DFT‐optimized structures is a well‐known phenomenon in metal complexes compared to experimentally measured distances.^[^ ^17^, ^32^, ^33^, ^34^ ^]^

**Figure 4:**
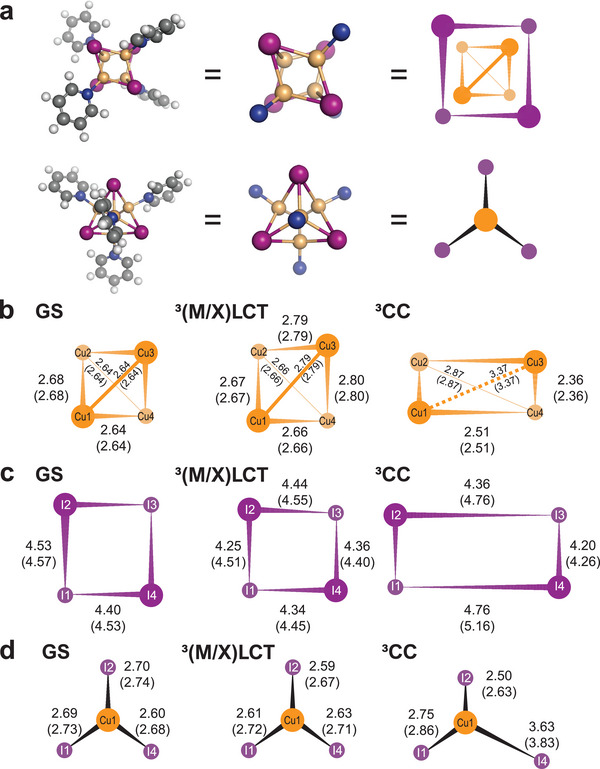
Refined molecular structures for GS, ^3^(M/X)LCT and ^3^CC states. a) Schematic diagram displaying the molecular structure of Cu_4_I_4_(py)_4_ (left), with pyridine ligands omitted for clarity (middle). On the right, a further simplified schematic diagram abstracts the representation for streamlined visualization. In this representation, atoms closer to the viewer are rendered larger and clearer, while those further away are smaller and more translucent. The images at the top and bottom each present the same structure from different perspectives for comprehensive visualization. b−d) Projections of the Cu_4_, I_4_ cores, and Cu−I_3_ framework in GS (left), ^3^(M/X)LCT (middle), and ^3^CC (right) states, exaggerated to highlight distinctive atomic pair distances. Here, the atomic pair distances obtained from DFT calculations are shown in parentheses, while those obtained from TRXL experiments are presented without parentheses. b,c) Structures of Cu_4_ (b) and I_4_ (c) cores for GS, ^3^(M/X)LCT, and ^3^CC states, viewed from the same direction as the top illustration in (a). For a clear description of the distortion in the Cu_4_ core in the ^3^CC state, the four Cu atoms in (b) are designated as follows: the atom in the lower left corner is labeled Cu1, with the subsequent atoms named clockwise as Cu2, Cu3, and Cu4, respectively. Likewise, the four I atoms in (c) are labeled in the same way as I1, I2, I3, and I4. In (b), the distances within 2.98 Å are depicted with solid lines, reflecting a reported Cu–Cu binding energy of −0.175 eV within that range.^[^ ^35^ ^]^ Distances beyond this threshold are indicated with dotted lines. d) Structures of the Cu−I_3_ framework for GS, ^3^(M/X)LCT, and ^3^CC states, viewed from the same direction as the lower illustration in (a). The illustration depicts the Cu1 atom and its adjacent three I atoms, namely I1, I2, and I4.

The 3D representations of refined structures and their corresponding structural parameters are depicted in Figure S15 (Supporting Information). We note that the Cu_4_I_4_(py)_4_ cluster has a complex structure, making the 3D representation challenging to understand at a glance. Therefore, in **Figure** **4**, we depicted an exaggerated and simplified schematic representation of the molecular structures to emphasize the symmetry of each structure and the differences between structures. The comparison between the theoretical difference scattering curves derived from these refined structures and the experimental DADS*_i_* (*q*)s is illustrated in Figure 3a. Meanwhile, once each DADS*_i_* (*q*) is assigned to transitions between specific species, we can extract the difference scattering curves for each species and track their concentration changes. Figure 3c shows the concentration profiles of the two species, ^3^(M/X)LCT and ^3^CC, over time.

### 2.4 Molecular Structures of the Ground State and the Two Excited States Observed via TRXL

In the case of GS, the Cu_4_I_4_ frame exhibits a high degree of symmetry with symmetrical structures for the Cu_4_ core. Specifically, in the GS of the Cu_4_I_4_(py)_4_ cluster, the six Cu–Cu interatomic distances are similar to each other, and the six I–I interatomic distances are also similar to each other. Figure 4b and Figure S15a (Supporting Information) showcase the spatial arrangement of Cu atoms in the GS, revealing a unique spatial pattern: Cu1 and Cu3 are positioned on an “upper floor”, relatively closer to the viewer when seen from a direction perpendicular to the plane of the figure. Conversely, Cu2 and Cu4, the remaining atoms, are located in the “lower floor”, farther from the viewer. As previously noted, the transition from GS to ^3^(M/X)LCT entails minimal structural changes. Comparisons shown in Figure 4b–d and Figure S15a,b (Supporting Information) reveal that changes in interatomic distances largely fall within a range of 0.2 Å. Specifically, ^3^(M/X)LCT exhibits a slight expansion in Cu–Cu distances alongside a slight contraction in I–I distances. This transition to the ^3^(M/X)LCT state results in a reduction in symmetry, manifesting as a broad distribution of both Cu–Cu and I–I interatomic distances within this state. The variation in Cu–Cu distances is attributed to the displacement of one Cu atom by ≈0.15 Å during the transition, whereas the positions of the other three Cu atoms remain largely unchanged from the GS.

In contrast to ^3^(M/X)LCT, the ^3^CC structure exhibits pronounced structural deviation from the GS structure. First and foremost, there is a marked reduction in the symmetry of the structure, a stark contrast to the minor reduction observed in the ^3^(M/X)LCT structure. In the ^3^CC structure, substantial positional shifts of Cu atoms relative to the GS are observed. For example, with Cu1 as reference, Cu2 and Cu4 shift significantly towards Cu1 (*r*(Cu1–Cu2): from 2.68 to 2.36 Å; *r*(Cu1–Cu4): from 2.64 Å to 2.51 Å), whereas Cu3 shifts significantly away from Cu1 (*r*(Cu1–Cu3): from 2.64 to 3.37 Å), indicating marked structural rearrangements. The longest Cu–Cu distance, ≈3.4 Å, is noteworthy because it exceeds twice the van der Waals radius, which is ≈2.8 Å. This extended distance suggests the loss of bonding interactions between the two Cu atoms. These structural changes lead to a significant alteration in the shape of the frame formed by the four Cu atoms (Figure 4b,c). In the GS structure, the four Cu atoms form a nearly regular tetrahedral shape. In the ^3^CC structure, this shape becomes highly distorted. The distortion is readily observable in the molecular structure even without exaggerated representation, as depicted in Figure S15 (Supporting Information), and is further highlighted in Figure 4b.

If we imagine connecting the Cu atoms in the same plane with lines—specifically, lines Cu1–Cu3 and Cu2–Cu4—in the GS and ^3^(M/X)LCT structures, these two lines are oriented nearly perpendicular (90°) to each other. In contrast, in the ^3^CC structure, the intersection angle of the two lines is markedly reduced to ≈52°. This pronounced distortion results in the ^3^CC structure exhibiting notably broad distributions of both Cu–Cu and I–I distances. The observed structural change in the ^3^CC structure is notable for its considerable reduction in symmetry and distortion of the Cu framework, signifying a notable deviation from a previous report.^[^ ^14^ ^]^ Although the presence of the ^3^CC state has been identified in other copper halides across both solid and solution phases, as well as in various functional materials incorporating copper halides as secondary building units,^[^ ^36^, ^37^ ^]^ such significant structural changes have not been suggested before. The interatomic distances of GS, ^3^(M/X)LCT, and ^3^CC states, obtained from the structural fit, are listed in Tables S6 and S7 (Supporting Information).

In a previous theoretical study on the excited state structures of Cu_4_I_4_(py)_4_, it was assumed that both emissive states—the ^3^CC state and the ^3^(M/X)LCT states—would maintain the D_2d_ symmetry observed in the GS.^[^ ^14^ ^]^ Contrary to this, our TRXL data exhibits better agreement with structures exhibiting reduced symmetry. Attempting to fit the excited‐state structures with the D_2d_ symmetry, as shown in Figure S16 (Supporting Information), fails to provide an adequate description of the experimental data, resulting in a χ_ν_^2^ value significantly higher (more than double) than that for structures with lowered symmetry. This observation is further supported by our TD‐DFT calculations. Our calculations indicate that the GS, ^3^CC and ^3^(M/X)LCT states possess C_1_ symmetry, displaying solely the identity element of symmetry. This observation challenges previous assumptions of D_2d_ symmetry for both the GS and the two emissive states. Specifically focusing on the Cu_4_I_4_ framework, while the GS showcases slightly higher symmetry S_4_, the ^3^CC and ^3^(M/X)LCT states display C_2_ and C_1_ symmetry, respectively. Taking these observations into consideration, we propose that the symmetry‐lowered structures, as derived from our theoretical calculations and further refined through our TRXL data, more accurately reflect the real structures of Cu_4_I_4_(py)_4_ in its excited states compared to those reported in a previous theoretical study.^[^ ^14^ ^]^

The distortion observed in the ^3^CC state may seem entirely attributable to a simple Jahn‐Teller effect. As shown in Figure S1 (Supporting Information) and confirmed by natural transition orbital (NTO) calculations, the electronic transitions leading to the formation of the ^3^CC state (S_7_, S_8_, and S_9_) predominantly involve transitions from the HOMO, HOMO‐1, and HOMO‐2 to the LUMO+4. Notably, HOMO‐1 and HOMO‐2 are degenerate, indicating the potential for Jahn‐Teller distortion due to unequally occupied degenerate orbitals.

However, two key observations indicate that the Jahn‐Teller effect alone cannot account for the substantial structural distortion in the ^3^CC state. First, both the ^3^CC and ^3^(M/X)LCT states involve degenerate orbitals in the electronic transitions that lead to their formation. If the Jahn‐Teller effect were solely responsible for the structural distortion, a similar degree of distortion would be expected in the ^3^(M/X)LCT state. However, the ^3^(M/X)LCT state shows no such significant distortion, suggesting that additional factors must contribute to the structural distortion observed in the ^3^CC state. Second, the molecular orbitals of Cu_4_I_4_(py)_4_ are composed of numerous closely packed orbitals due to the presence of the four copper and iodine atoms. Consequently, the structural changes in the excited state of Cu_4_I_4_(py)_4_ are likely influenced by interactions between these closely stacked orbitals, complicating the interpretation of the distortion as a result of a single phenomenon.

### 2.5 Overall Structural Dynamics of Cu_4_I_4_(py)_4_

**Figure 5:**
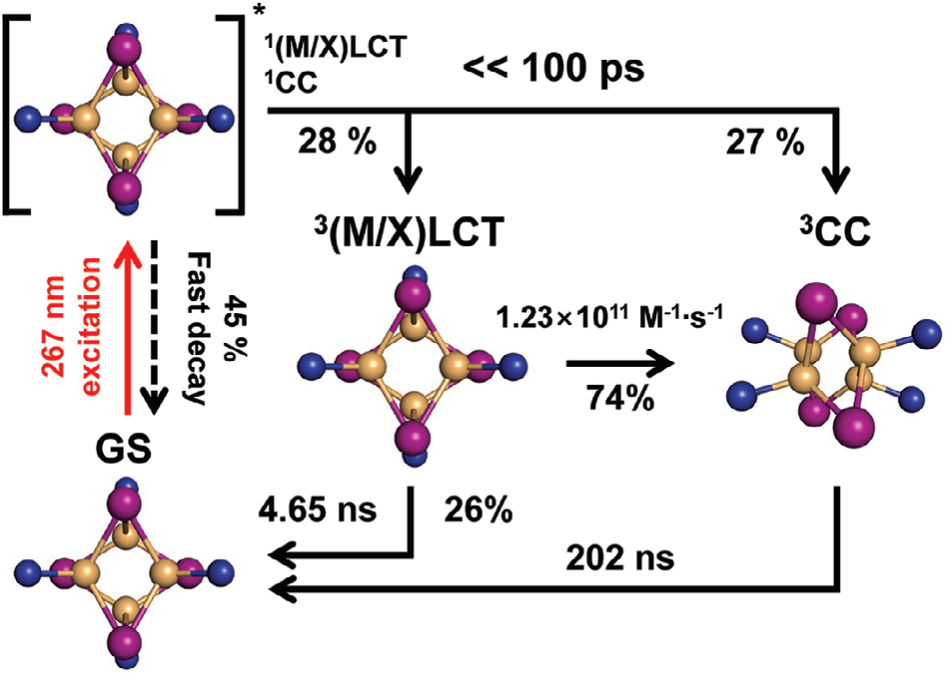
Proposed dynamics of Cu_4_I_4_(py)_4_. The 267 nm pump pulse initially excites Cu_4_I_4_(py)_4_ into the ^1^(M/X)LCT and ^1^CC states, based on the result of the TD‐DFT calculations. Then, 45% of the excited molecules rapidly decay to the ground state, releasing excess energy as heat. Of the remaining 55%, 28% populate the ^3^(M/X)LCT state within 100 ps, while 27% populate the ^3^CC state within the same period. 74% of the^3^(M/X)LCT state undergoes a transition to the ^3^CC state with a bimolecular rate constant of 1.23 × 10^11^ m ^−1^∙s^−1^, while the remaining 26% of the state relaxes to the GS. Subsequently, molecules in the ^3^CC state recover to the ground state with a time constant of 202 ns.

The overall structural dynamics of Cu_4_I_4_(py)_4_ upon photoexcitation at 267 nm, including the molecular structures of the excited states and GS, are depicted in **Figure** **5**. This scheme provides a detailed and comprehensive view of the pathways and transitions that occur following photoexcitation of Cu_4_I_4_(py)_4_. Initially, 0.524 mm out of the total 5 mm of Cu_4_I_4_(py)_4_ molecules in the GS undergo excitation, initiating a series of relaxation and energy transfer processes. Of these photoexcited molecules, ≈45% relax rapidly back to the GS, releasing their excess energy as heat into the surrounding solvent. This relaxation process occurs on a timescale too fast to resolve within the IRF of this experiment. The remaining 55% of the photoexcited molecules partition into the ^3^(M/X)LCT and ^3^CC states in a ratio of 51:49 within 100 ps. The ^3^(M/X)LCT state, after 100 ps, exhibits two competing decay pathways. The major pathway involves a transition to the ^3^CC state, while the minor pathway corresponds to a direct decay to the GS. The major pathway, the transition to the ^3^CC state, is hypothesized to occur via a bimolecular energy transfer mechanism in which energy is transferred from a molecule in the ^3^(M/X)LCT state to another molecule in the GS. From our TCSPC measurements, the bimolecular rate constant for this process is determined to be 1.43×10^11^ M^−1^ s^−1^. In the minor pathway, the decay to the GS occurs independently of the concentration of Cu_4_I_4_(py)_4_, with a rate constant of 2.51 × 10^8^ s^−1^. Under the experimental condition of 5 mm used in our TRXL measurements, the relative contributions of these two transitions, ^3^(M/X)LCT → ^3^CC and ^3^(M/X)LCT → GS, are 74:26. Assuming that the same ratio applies to the observed apparent time constant of 1.21 ns from the TRXL measurements, we calculated the rate constants for each transition. The rate constant for the ^3^(M/X)LCT → ^3^CC transition was determined to be 1.23 ± 0.83 × 10^11^ m ^−1^·s^−1^, while the rate constant for the ^3^(M/X)LCT → GS transition was calculated to be 2.15 × 10^8^ s^−1^, which corresponds to a time constant of 4.65 ± 2.35 ns. Following the transition to the ^3^CC state, this state decays back to the GS with a time constant of 202 ± 37 ns. This final step represents the complete recovery of the system to its ground state after photoexcitation. We note that the initial population of the two distinct states, ^3^(M/X)LCT and ^3^CC, could not be resolved due to the temporal resolution limit of the current experiment, which is ≈100 ps. To capture this early‐stage dynamic process, further investigation using TRXL with femtosecond temporal resolution (fs‐TRXL) is necessary. Such measurements would provide valuable insights into the ultrafast process that govern the population of these states.

## 3 Conclusion

By taking advantage of the high sensitivity of TRXL experiments to the structural details of molecules with heavy atoms, we successfully tracked the time‐dependent structural changes of metal‐halide cubane clusters triggered by UV excitation, which had not been previously explored experimentally. Our experiments and subsequent data analysis elucidated the following insights: i) Initially, within a time delay of 100 ps, Cu_4_I_4_(py)_4_ populates two distinct excited states: the ^3^(M/X)LCT state and the ^3^CC state. ii) The system undergoes a transition from the ^3^(M/X)LCT state to the ^3^CC state, followed by a recovery of the ^3^CC state back to the GS. Notably, the ^3^CC state exhibited significant structural deviations from the ground state, especially evident in the marked symmetry reduction characterized by a pronounced tilt between the upper and lower Cu_2_I_2_ layers within the Cu_4_I_4_ framework. Such observations might also be relevant to other cubane‐type metal halide complexes that exhibit the ^3^CC state. In contrast, the ^3^(M/X)LCT did not show significant structural changes compared to the dramatic alterations observed in the ^3^CC state. These insights enrich our understanding of cubane‐type metal cluster compounds, opening avenues for their potential applications, including their use as soft secondary building units in metal–organic frameworks. We believe this study represents a significant milestone in the field, as it is the first to experimentally investigate the detailed structural dynamics of cubane‐type metal complexes following photoexcitation.^[^ ^38^, ^39^, ^40^, ^41^, ^42^, ^43^, ^44^, ^45^, ^46^, ^47^, ^48^, ^49^, ^50^, ^51^, ^52^, ^53^, ^54^, ^55^, ^56^, ^57^, ^58^, ^59^, ^60^ ^]^

## Conflict of Interest

The authors declare no conflict of interest.

## Supporting information



Supporting Information

## Data Availability

The data that support the findings of this study are available from the corresponding author upon reasonable request.
