# Supplementary material for: Excited‐State Structural Dynamics of the Cubane‐Type Metal Cluster [Cu4I4(py)4] Explored by Time‐Resolved X‐Ray Liquidography
Source: Adv Sci (Weinh). 2025 Feb 14;12(14):2414970. doi: 10.1002/advs.202414970 (PMC11984917; doi:10.1002/advs.202414970)
Supplement: Supplementary file 1 — Supporting Information [file ADVS-12-2414970-s001.pdf]

ADVANCED SCIENCE
Open Access

Supporting Information

for *Adv. Sci.*, DOI 10.1002/advs.202414970

Excited-State Structural Dynamics of the Cubane-Type Metal Cluster $[Cu_4I_4(py)_4]$ Explored by Time-Resolved X-Ray Liquidography

*Doyeong Kim, Hosung Ki, Donghwan Im, Yunbeom Lee, Seonggon Lee, Jun Heo, Seunghwan Eom, Eun Hyuk Choi, Doo-Sik Ahn and Hyotcherl Ihee**

SUPPORTING INFORMATION

Excited-state structural dynamics of the cubane-type metal cluster [$Cu_4I_4(py)_4$] explored by time-resolved X-ray liquidography

Doyeong Kim,[a,b] Hosung Ki,[a,b] Donghwan Im,[a,b] Yunbeom Lee,[a,b] Seonggon Lee,[a,b] Jun Heo,[b] Seunghwan Eom,[a,b] Eun Hyuk Choi,[a,b] Doo-Sik Ahn,[b] and Hyotcherl Ihee [a,b]*

[a]Department of Chemistry, Korea Advanced Institute of Science and Technology (KAIST), Daejeon, 34141, Republic of Korea

[b]Center for Advanced Reaction Dynamics, Institute for Basic Science (IBS), Daejeon 34141, Republic of Korea

Cubane-type metal cluster, reaction dynamics, structural dynamics, time-resolved X-ray liquidography, reaction intermediate

E-mail: hyotcherl.ihee@kaist.ac.kr

Supporting Methods

Time-resolved X-ray liquidography (TRXL) experiment

Time-resolved X-ray liquidography (TRXL), also known as time-resolved X-ray solution scattering, is a pump-probe experimental technique that uses an optical laser as the pump and X-rays as the probe. This method analyzes the scattering pattern resulting from constructive and destructive interference as X-rays scatter from a solution-phase sample, based on the interatomic distances within the molecules. By interpreting these patterns, TRXL allows the observation of structural changes occurring in solution. The X-ray diffraction intensity is typically expressed as a function of q, the magnitude of the momentum transfer between the incident and the elastically scattered X-ray waves. In detail, q is given by $q = (4\pi/\lambda)\sin(2\theta/2)$, where 2θ and λ denote the scattering angle and X-ray wavelength, respectively. The scattering pattern obtained from the TRXL experiment includes both elastic and inelastic scattering. Since we deal with the difference scattering signal, most of the inelastic scattering, which is unrelated to molecular structure, is canceled out in the difference signal. The elastic scattering signal from the solution in the TRXL experiment can be divided into three terms: a solute term, a solute-solvent term (also known as "cage" term), and a solvent term. First, the solute term is calculated using the atomic form factors (f_i and $f_{j,}$) and the interatomic distances (r_{ij}) within each solute molecule. This calculation is performed using the Debye equation, as shown below.

$$\mathrm{S}_{solute}(q) = \sum_i f_i(q)^2 + \sum_i \sum_{j \neq i} f_i(q) f_j(q) \frac{\sin(qr_{ij})}{qr_{ij}} \quad \text{(S1)}$$

The cage term describes the signal component resulting from the interference between two waves, each scattered by a solute atom and a solvent atom, respectively. It is calculated using pair distribution functions (PDFs), $g_{ij}(r)$, which represent the distribution of distances between atoms. Here, i indexes atom types, or elements, of the atoms belonging to the solute molecule, and j indexes atom types, or elements, of the atoms belonging to the solvent molecule. These PDFs are extracted from molecular dynamics (MD) snapshots. The cage term is then calculated using the following equation.

$$\mathrm{S}_{cage}(q) = \sum_i^{solute} \sum_j^{solvent} \frac{N_i N_j}{V} f_i(q) f_j(q) \int_0^\infty 4\pi r^2 (g_{ij}(r) - 1) \frac{\sin(qr_{ij})}{qr_{ij}} dr \quad \text{(S2)}$$

Here, N_i, N_j, and V represent the number of atoms corresponding to the i-th and j-th atom types, and the number of atoms contained in the overall volume of the virtual MD box, respectively.

Lastly, the solvent term is well-characterized, with its profile—referring to the shape of the solvent term in q-space—is relatively well-known and can be determined through a separate experiment, as documented in various studies.[1-3] In this study, we obtained this profile through an additional TRXL experiment using a 4-bromo-4′-(*N*,*N*-diethylamino)-azobenzene dye solution, which is known for efficiently transferring heat to solvent molecules without undergoing structural changes upon UV irradiation.

The TRXL experiments were conducted at beamline ID09 of the European Synchrotron Radiation Facility (ESRF). A typical TRXL setup, employing an optical femtosecond laser to generate the pump pulses and X-ray as the probe, was utilized.[4] The 800 nm wavelength from the fundamental output of a 1-kHz amplified Ti:sapphire laser system was converted to 267 nm through frequency tripling. The tripled optical laser pulses, delivering ~31 μJ/pulse at the sample position, were focused to an area of 180 × 190 μm^2, resulting in a fluence of 1.1 mJ/mm^2. Following photoexcitation by a laser pulse, the scattering of an X-ray pulse probed the structural changes driven by the photoreaction. A pink X-ray beam used as the X-ray probe was generated by the passage of electrons through the U17 undulator and had a center energy of 17.57 keV with a finite bandwidth of ΔE/E = ~4%, a pulse duration of 100 ps, and delivered approximately 5 × 10^8 photons per pulse.[5] The scattered

X-ray photons by the sample solution were collected by an area detector (Rayonix MX170-HS, 1920 × 1920 pixels, 89 mm pixel size) in 2 × 2 binning mode with a sample-to-detector distance of 42 mm and an exposure time of three seconds per image. The sample solution of 5.0 mM $Cu_4I_4(py)_4$ (Samchun chemical, synthesized) in acetonitrile (Sigma-Aldrich, anhydrous, 99.8%) was circulated through a quartz capillary nozzle of 300 μm thickness. This open jet system provided a stable flow of liquid with a speed fast enough to deliver fresh sample solutions to the interaction position of X-ray and laser for every pump-probe measurement. We acquired the solution scattering data at the pump-probe time delays of $t = -3$ ns, 100 ps, 178 ps, 316 ps, 562 ps, 1 ns, 1.78 ns, 3.16 ns, 5.62 ns, 10 ns, 17.8 ns, 31.6 ns, 56.2 ns, 100 ns, 178 ns, and 562 ns. In the TRXL experiment designed to obtain the solvent term, a solution of 4-bromo-4'-(N,N-diethylamino)-azobenzene dye was prepared at a concentration of 0.833 mM. The TRXL experiment on the dye solution was conducted under two fluence conditions: 1.1 mJ/mm^2 and 1.6 mJ/mm^2. For the 1.1 mJ/mm^2 condition, data were collected at −3 ns, 100 ps, and 1 μs, while for the 1.6 mJ/mm^2 condition, data were acquired at −3 ns, 100 ps, 1 ns, 10 ns, 100 ns, and 1 μs. Details on the processing of the TRXL data are provided in the "Data processing" section.

Data processing

The two-dimensional scattering images were azimuthally integrated as a function of the magnitude of the momentum transfer vector (q), represented by $q = (4\pi/\lambda)\sin(2\theta/2)$. Here, λ denotes the X-ray wavelength, and 2θ signifies the scattering angle. These scattering curves were normalized by their area within q values ranging from 4.0 to 8.0 $Å^{-1}$. Following normalization, the difference scattering curves, $\Delta S(q, t)$, were generated by subtracting the scattering curve at a negative time delay (t = −3 ns) from those at positive time delays.

The raw experimental data were initially expressed in arbitrary units, which lack standardization for quantitative interpretation. To extract quantitative information from the data, such as the concentration of photoexcited solute molecules or the changes in solvent temperature or density during the photoreaction, it is necessary to convert the data into a standard unit, such as electron unit per solvent molecule [ref]. For the purpose of unit conversion, two independent scaling parameters need to be determined. The first parameter (P_1) is the scaling factor between the theoretical static scattering curve ($S'(q)$), calculated using the Debye equation and expressed in electron unit per molecule, and the experimental static scattering curve ($S(q, t)$). The relation can be expressed as follows.

$$P_1 \cdot S(q,t) = S'(q) \tag{S3}$$

Determination of P_1 was achieved by comparing the theoretical scattering curve from the bulk solvent, $S'_{solvent}(q)$, with its experimental homologue, $S_{solvent}(q, t)$. For the calculation of $S'_{solvent}(q)$ from the bulk solvent, we utilized the PDFs obtained from MD simulations of the bulk solvent. Using the PDF, we first calculated $S'_{solvent}(q)$ corresponding to the entire unit cell of the MD simulation. Subsequently, $S'_{solvent}(q)$ was normalized by dividing it by the number of solvent molecules in the unit cell, yielding $S'_{solvent}(q)$ corresponding to a single solvent molecule. This single-molecular $S'_{solvent}(q)$ was then compared with $S_{solvent}(q, t)$. However, while $S'_{solvent}$ only considers elastic scattering contributions from the solvent, the experimental data includes air or incoherent scattering contributions as well, thereby making a direct comparison between $S_{solvent}(q, t)$ and $S'_{solvent}(q)$ challenging. These unintended scattering contributions can be wiped out by taking the difference between two static scattering curves or taking the derivative of a static scattering curve with respect to q. Thus, we compared the derivatives of the two terms, $dS_{solvent}(q, t)/dq$ and $dS'_{solvent}(q)/dq$. The corresponding equation is provided below:

$$P_1 \cdot dS(q,t) / dq = dS'(q) / dq \tag{S4}$$

The second parameter (P_2) is calculated by determining the scaling factor between the experimental static scattering curve and the experimental difference scattering curve ($\Delta S(q, t)$). The equation for the second parameter is given by:

$$P_2 \cdot \Delta S(q,t) = (S(q,t) - S(q,t_{ref})) \tag{S5}$$

where t_{ref} is −3 ns. As mentioned earlier, when generating the difference scattering curve, both $S(q, t)$ and $S(q, t_{ref})$ are normalized before subtraction to yield the difference scattering curve. The relationship between $S(q, t)$ and $\Delta S(q, t)$ is defined as follows:

$$\Delta S(q,t) = (S(q,t) / v(t) - S(q,t_{ref}) / v(t_{ref})) \tag{S6}$$

where $v(t)$ denotes the normalization factor of the static experimental scattering curve at the time delay t. Under typical experimental conditions, $v(t_{ref}) \approx v(t) \approx v$, allowing the equation to be simplified:

$$\Delta S(q,t) = (S(q,t) / v(t) - S(q,t_{ref}) / v(t_{ref})) \approx (S(q,t) / v - S(q,t_{ref}) / v) = 1 / v \cdot (S(q,t) - S(q,t_{ref})) \tag{S7}$$

where v represents the average normalization factor of the static experimental scattering curve. Comparing Equation S7 with Equation S5 leads to the relation: $P_2 = 1/v$. This means P_2 can be directly determined by taking the inverse of the average of the normalization factor of the static experimental scattering curves used for generating the difference scattering curve.

Upon determining P_1 and P_2, the experimental difference scattering curves were converted into electron unit per molecule using the following equation:

$$\begin{aligned} \Delta S(q,t) &= 1 / P_2 \cdot (S(q,t) - S(q,t_{ref})) \\ &= 1 / P_2 \cdot 1 / P_1 \cdot \sum_i^N (c_i(t) \cdot (S'_i(q) - S'_{react}(q)) \end{aligned} \tag{S8}$$

$$\Delta S_{eu}(q,t) = P_1 \cdot P_2 \cdot \Delta S(q,t) = \sum_i^N (c_i(t) \cdot (S'_i(q) - S'_{react}(q))$$

where N is the number of transient species present at the time delay t, $c_i(t)$ is the molar ratio of the i-th transient species relative to the solvent present at the time delay t, $\Delta S'_i(q)$ is the theoretical difference scattering curve corresponding to the i-th transient species, $S'_i(q)$ and $S'_{react}(q)$ are the theoretical scattering curves corresponding to the i-th transient species and reactant, respectively, and $\Delta S_{eu}(q, t)$ represents the experimental difference scattering curve expressed in the unit of electron unit per molecule. In this equation, as both $S'_i(q)$ and $S'_{react}(q)$ are in electron unit per molecule, we can convert the raw experimental data, $\Delta S(q, t)$, to the electron unit per molecule by multiplying by the factor of $P_1 \cdot P_2$.

During our experiments, we measured the solvent heating signal under two different laser fluence conditions (1.1 mJ/mm^2 and 1.6 mJ/mm^2) to investigate potential artifacts arising from high laser fluence. Upon comparing the difference scattering curves obtained under these conditions, we observed noticeable differences in their shapes (Figure S5), suggesting the presence of an additional artifact component beyond the expected solvent contributions, such as the $(\partial S/\partial T)_\rho$ and $(\partial S/\partial \rho)_T$ terms. This artifact likely arises from an abnormal solvent response induced by high laser fluence, though the precise mechanism remains unclear.

To account for this artifact, we extracted the artifact component from the data measured at the higher laser fluence (1.6 mJ/mm^2). First, we processed the TRXL data at the lower laser fluence (1.1 mJ/mm^2) using established methods to convert the scattering signals into the $(\partial S/\partial T)_\rho$ and $(\partial S/\partial \rho)_T$ terms.[6-7] Then, we applied the Projection to extract the perpendicular component (PEPC) method to the first left singular vector derived from the SVD analysis of the 1.6 mJ/mm^2 data (purple curve in Figure S5a), using the $(\partial S/\partial T)_\rho$ and $(\partial S/\partial \rho)_T$ obtained from the 1.1 mJ/mm^2 data (red and blue curves in Figure S5a), isolating the component orthogonal to the $(\partial S/\partial T)_\rho$ and $(\partial S/\partial \rho)_T$ terms from the 1.1 mJ/mm^2 data. This orthogonal component represents the artifact associated with the high laser fluence (purple curve in Figure S5b).

When applying the PEPC method to extract the solute-only kinetics of $Cu_4I_4(py)_4$, we incorporated this artifact component (purple curve in Figure S5b), along with the $(\partial S/\partial T)_\rho$ (red curve in Figure S5b) and $(\partial S/\partial \rho)_T$ (blue curve in Figure S5b) terms derived from the 1.1 mJ/mm^2 data. This approach ensured that the solute-only kinetics were accurately determined, accounting for all solvent-related contributions, including those from the high-fluence artifact.

Projection to extract the perpendicular component (PEPC) method

The PEPC method removes solvent contributions from TRXL data, enabling precise analysis of solute-specific kinetics and structural dynamics by effectively isolating the solute-related signal, $\Delta S_{\text{solute-related}}$, where $\Delta S_{\text{solute-related}} = \Delta S_{\text{solute}} + \Delta S_{\text{cage}}$. At each time delay t, the TRXL signal, $\Delta S(q, t)$ can be treated as a vector in q-space. By applying vector projection, the PEPC method projects the TRXL signal onto a subspace orthogonal to the solvent terms. This subspace, referred to as the "solvent-contribution-free space", nullifies the contribution of the solvent term, effectively eliminating its influence on the TRXL signal. We denote the data projected into this space using the superscript ⊥ symbol. In this transformed space, the solvent contribution becomes negligible:

$$\Delta S^{\perp}_{\text{solvent}}(q,t) = 0, \tag{S9}$$

Thus, the PEPC-treated signal simplifies to:

$$\begin{aligned}\Delta S^{\perp}(q,t) &= (\Delta S(q,t)_{\text{solute}} + \Delta S(q,t)_{\text{cage}} + \Delta S(q,t)_{\text{solvent}})^{\perp} \\ &= \Delta S^{\perp}(q,t)_{\text{solute}} + \Delta S^{\perp}(q,t)_{\text{cage}} + \Delta S^{\perp}(q,t)_{\text{solvent}} \\ &= \Delta S^{\perp}(q,t)_{\text{solute}} + \Delta S^{\perp}(q,t)_{\text{cage}}\end{aligned} \tag{S10}$$

This ensures that the solvent influence is effectively screened out, leaving only the solute- and cage-related components for further analysis. We note that, in addition to removing solvent contributions, the PEPC method can also eliminate artifacts unrelated to the solute-related term, provided that the shapes of these artifacts are known. This is achieved by projecting the TRXL signal onto a subspace orthogonal to both the solvent terms and the known artifacts. A more detailed explanation of the PEPC method and its application can be found in the previous work.[8]

Since the solvent terms and artifacts are generally not orthogonal to the solute-related components, the shape of $\Delta S^{\perp}(q, t)$ in q-space may exhibit distortions compared to $\Delta S_{\text{solute-related}}$. These distortions are corrected through structural analysis, allowing the accurate retrieval of $\Delta S_{\text{solute-related}}$, as detailed in previous publications.[2, 9] Consequently, the PEPC method extracts the solute-related components, $\Delta S_{\text{solute-related}}$, and solvent components, $\Delta S_{\text{solvent}}(q, t)$, from the total TRXL signal, $\Delta S(q, t)$, providing a clear understanding of the solute's structural and kinetic behavior.

Singular value decomposition (SVD) analysis

To quantitatively analyze the experimental data for $Cu_4I_4(py)_4$, we employed the SVD analysis, which decomposes the data into time-independent signal components in q-space (left singular vectors, LSVs), their relative contributions (singular values), and their time-dependent behaviors (right singular vectors, RSVs). For the SVD analysis, we built an $n_q \times n_t$ matrix of the difference scattering curves, **A**, where n_q is the number of q points in the difference scattering curves and n_t is the number of time-delay points. Via SVD, the matrix **A** is decomposed into three matrices satisfying the relationship of $\mathbf{A} = \mathbf{USV}^{\mathrm{T}}$. **U** is an $n_q \times n_t$ matrix whose column vectors are LSVs of **A**, **V** is an $n_t \times n_t$ matrix whose column vectors are RSVs of **A**, and **S** is a diagonal $n_t \times n_t$ matrix whose diagonal elements are singular values of **A**. The matrices **U** and **V** follow the relationships of

$\mathbf{U}^T\mathbf{U} = \mathbf{I}_{nt}$ and $\mathbf{V}^T\mathbf{V} = \mathbf{I}_{nt}$, respectively, where $\mathbf{I}_{nt}$ is an identity matrix. The RSVs obtained from the SVD results were then utilized for the kinetic analysis. The number of the singular vectors, which significantly contribute to the signal, was determined from the SVD results based on the singular value and autocorrelation values.

Kinetic analysis

As previously mentioned, the TRXL signals consist of three components: (i) the solute term, (ii) the cage term, and (iii) the solvent term. Typically, the kinetics of the first two terms (solute and cage terms) directly reflect the kinetics of the solute molecules, whereas the last term (solvent term) can have different kinetics from those of the solute molecules. Considering that the kinetics of solute molecules are generally the primary focus, rather than those of solvent molecules, it is advantageous to isolate the solute-only kinetics by removing the kinetic contributions of the solvent molecule from the TRXL signals, thereby simplifying the analysis. For such a purpose, we applied the PEPC method, which was recently developed for this purpose, to our data.[8] By applying the PEPC method, we removed the kinetic contributions of the following three components: two heating components ($(\partial S/\partial T)_\rho$ and $(\partial S/\partial \rho)_T$ terms) and an artifact component caused by high laser fluence, all derived from a separate experiment with a dye solution. Subsequently, we performed SVD analysis on the PEPC-treated data $\Delta S^{\perp}(q, t)$, which we refer to as "solvent-contribution-free" data in the main text, to analyze the kinetics of the solute molecules. Using SVD, $\Delta S^{\perp}(q, t)$ was decomposed into LSVs, RSVs, and singular values. Figure S7a–c shows the LSVs, RSVs, and singular values, all obtained from the SVD analysis, along with the autocorrelation values of the LSVs and RSVs. Based on the autocorrelation values and the singular values, we concluded that two major components contribute significantly to the PEPC-treated experimental data. To extract the kinetic information, the first two RSVs were fitted with varying numbers of exponential functions. Using a single exponential, as illustrated in Figure S8a, fell short in accurately describing the observed time-dependent changes in the signal. Employing three exponentials, as demonstrated in Figure S8c, did not exhibit a significant improvement when compared to the results obtained using two exponentials. As a result, we concluded that the two RSVs are well described as a sum of a constant and two exponential functions, yielding time constants of 1.21 ns and 202 ns, respectively. We note that the constant term was fixed to zero for the fitting of the RSV1. The fitting results are shown in Figure S7d.

By using the two time constants obtained from the exponential fit of the RSVs, we extracted decay-associated difference scattering curves (DADSs) from the PEPC-treated data, $\Delta S^{\perp}(q, t)$. The resulting curves, DADS(q)s, represent the individual signal components in q-space, extracted from the experimental data. Each component is associated with a specific time constant, and its contribution to the overall time-resolved data follows the time profile of an exponential decay function characterized by that time constant. To extract DADS(q)s, we applied kinetics-constrained analysis (KCA). The term, DADS, is derived from "decay-associated spectra" (DAS), a concept widely used in the field of spectroscopy to represent spectra associated with individual decay time constants.[10] The process of extracting DADS can be mathematically described as follows. First, the relationship between the DADS and the TRXL data is established as follows:

$$\Delta S^{\perp}(q,t) = \sum \mathrm{DADS}_i(q) \cdot \exp(-t/\tau_i) \qquad \text{(S11)}$$

Here, $\Delta S^{\perp}(q, t)$ represents the PEPC-treated difference scattering curves, and $\mathrm{DADS}_i(q)$ corresponds to the DADS associated with the i-th time constant. As Equations S11 show, the concept of DADS is identical to that of DAS used in spectroscopy. The only difference lies in the data from which they are extracted: spectra for DAS and difference scattering curves for DADS. The underlying principle for extracting DADSs from $\Delta S^{\perp}(q, t)$ can be described through the following mathematical equations. The PEPC-treated data, $\Delta S^{\perp}(q, t)$, can be expressed as a linear combination of the DADSs, weighted by their corresponding relative contributions, or weights, as follows:

$$\Delta S^{\perp}(q,t) = \sum \text{DADS}_i(q) \cdot \text{c}_i(t) \tag{S12}$$

Here, $c_i(t)$ is the relative contribution of the i-th DADS at the time delay t. Ideally, $c_i(t)$ follows the exponential decay function $\exp(-t/\tau_i)$ as shown in Equation S11. Equation S12 can be rewritten in matrix form as follows:

$$\begin{aligned}
\Delta S^{\perp}(q,t) &= \left[\Delta S^{\perp}(q,t_1), \Delta S^{\perp}(q,t_2), \ldots, \Delta S^{\perp}(q,t_{n_i})\right] \\
&= \left[\sum \text{DADS}_i(q) \cdot \text{c}_i(t_1), \sum \text{DADS}_i(q) \cdot \text{c}_i(t_2), \ldots, \sum \text{DADS}_i(q) \cdot \text{c}_i(t_{n_i})\right] \\
&= \left[\text{DADS}_1(q), \text{DADS}_2(q), \ldots, \text{DADS}_{n_i}(q)\right] \cdot \left[\text{c}_i(t_1), \text{c}_i(t_2), \ldots, \text{c}_i(t_{n_i})\right]^{\text{T}} \\
&= \text{M}(q)\text{C}(t)^{\text{T}}
\end{aligned} \tag{S13}$$

where n_i is the number of time constants (in this case, two), M(q) is a matrix containing the $\text{DADS}_i(q)$ components, corresponding to the first term on the right side of the third line in Equation S13. Similarly, C(t) is a matrix representing the time profiles of the relative contributions, corresponding to the second term on the right side in the same line. Transposing Equation S13 results in the following equation in the well-known form of AX = B.

$$\text{C}(t)\text{M}(q)^{\text{T}} = (\Delta S^{\perp}(q,t))^{\text{T}} \tag{S14}$$

The $\text{DADS}_i(q)$s can be obtained by solving Equation S14 for M(q). Since the other two terms, C(t) and $\Delta S^{\perp}(q, t)$, are already known, the method for solving Equation S14 is straightforward and can be explained in detail as follows. To calculate M(q), we calculated the Moore-Penrose inverse, in other words, pseudoinverse, of the matrix C(t) and multiplied its transpose to $\Delta S^{\perp}(q, t)$. The pseudoinverse is mathematically expressed as follows:

$$\text{C}(t)^{+} = (\text{C}(t)^{\text{T}}\text{C}(t))^{-1} \tag{S15}$$

With the pseudoinverse of C(t), the DADS(q) curves can be obtained by using the following equation.

$$\begin{aligned}
\left[\text{DADS}_1(q), \text{DADS}_2(q), \ldots, \text{DADS}_{n_i}(q)\right] = \text{M}(q) &= \left\{\text{C}(t)^{+}(\Delta S^{\perp}(q,t))^{\text{T}}\right\}^{\text{T}} \\
&= \Delta S^{\perp}(q,t)(\text{C}(t)^{+})^{\text{T}}
\end{aligned} \tag{S16}$$

We conducted structural analysis on the obtained DADSs to identify the structural process corresponding to each DADSs, or each time constant. Based on the structural analysis results, we extracted the detailed structural information for each species.

Structure refinement by DADS fitting

Structure refinement using TRXL data involves fitting theoretical scattering curves generated from postulated model structures to experimental difference scattering curves. Specifically, this process refines the model structures to minimize the discrepancy between their theoretical DADS and the experimental DADS obtained through the kinetic analysis of PEPC-treated TRXL data. Additional details on the kinetic analysis and the PEPC method are provided in the sections titled "Projection to extract the perpendicular component (PEPC) method" and "Kinetic analysis" in SI. The DADS(q), derived from the PEPC-treated data $\Delta S^{\perp}(q, t)$, is free from contributions of solvent term and other artifact contributions. Therefore, it exclusively represents the solute-related contributions, comprising the sum of the solute term and the cage term. In other words, theoretical DADS, DADS′(q), can be expressed by the following equation:

$$\text{DADS}'(q) = \text{DADS}'_{\text{solute}}(q) + \text{DADS}'_{\text{cage}}(q) \tag{S17}$$

where $\text{DADS}'_{\text{solute}}(q)$ and $\text{DADS}'_{\text{cage}}(q)$ refer to the solute and cage terms, respectively, and their sum constitutes DADS′(q).

The $DADS'_{solute}(q)$ and $DADS'_{cage}(q)$ can be further described as the differences between the static terms, $S'_{solute}(q)$ and $S'_{cage}(q)$, respectively, corresponding to the specific transition associated with the DADS. When the transition corresponding to the i-th theoretical DADS, $DADS'_i(q)$, is A → B, the mathematical expression for the $DADS'_i(q)$ can be expressed as follows:

$$\mathrm{DADS}'_i(q) = c_i \cdot \left\{ \left(\mathrm{S}'_{\mathrm{A,\,solute}}(q) - \mathrm{S}'_{\mathrm{B,\,solute}}(q) \right) + \left(\mathrm{S}'_{\mathrm{A,\,cage}}(q) - \mathrm{S}'_{\mathrm{B,\,cage}}(q) \right) \right\} \quad \text{(S18)}$$

where c_i is the molar ratio of species A, which participates in the A → B transition corresponding to the $DADS'_i(q)$, $S'_{A,\,solute}(q)$ and $S'_{B,\,solute}(q)$ are the theoretical solute terms for species A and B, respectively, and $S'_{A,\,cage}(q)$ and $S'_{B,\,cage}(q)$ are the theoretical cage terms for species A and B, respectively. If multiple transitions contribute simultaneously to $DADS'_i(q)$, the Equation S18 can be modified as follows. Let the transitions involved in $DADS'_i(q)$ be represented as $A_j \rightarrow B_j$, where $j = 1, 2, \ldots, N$ and N is the number of transitions contributing to $DADS'_i(q)$. In this case, the equation for $DADS'_i(q)$ can be expressed as:

$$\mathrm{DADS}'_i(q) = \sum_{j=1}^{N} \left[c_{ij} \cdot \left\{ \left(\mathrm{S}'_{\mathrm{A}j,\,\mathrm{solute}}(q) - \mathrm{S}'_{\mathrm{B}j,\,\mathrm{solute}}(q) \right) + \left(\mathrm{S}'_{\mathrm{A}j,\,\mathrm{cage}}(q) - \mathrm{S}'_{\mathrm{A}j,\,\mathrm{cage}}(q) \right) \right\} \right] \quad \text{(S19)}$$

where c_{ij} is the molar ratio of species A_j, which participates in the $A_j \rightarrow B_j$ transition corresponding to the $DADS'_i(q)$, $S'_{Aj,\,solute}(q)$ and $S'_{Bj,\,solute}(q)$ are the theoretical solute terms for species A_j and B_j, respectively, and $S'_{Aj,\,cage}(q)$ and $S'_{Bj,\,cage}(q)$ are the theoretical cage terms for species A_j and B_j, respectively. Using Equation S19, $DADS'_i(q)$ was calculated based on the structures of the states identified to be involved in the transition corresponding to each DADS.

We note that the "pristine" theoretical DADSs are not suitable for direct comparison with experimentally obtained $DADS_i(q)$s for the following two reasons: i) the energy profile of the X-ray beam used in the experiment was polychromatic, having a broad bandwidth of $\Delta E/E$ = ~4%; ii) the experimental DADSs were PEPC-treated. To address these issues, we corrected the theoretical DADSs for a proper comparison. Firstly, we employed an established protocol to take into account the influence of the energy spectrum of the X-ray into the theoretical DADSs.[4] Subsequently, we performed PEPC on the theoretical DADSs using the same components used for the experimental data to remove the contributions of the solvent terms.[8] The theoretical DADS used during the structural fitting analysis is obtained in this manner.

Since $DADS'_i(q)$ was calculated based on the model structures, $DADS'_i(q)$s can be described as a function of the corresponding molecular structures. Therefore, optimal molecular structures must exist that best describe the experimentally obtained $DADS_i(q)$. Here, the process of identifying the optimal molecular structure is referred to as structure refinement. During the structure refinement, we parameterized and adjusted the model structures to find the optimal configuration that most accurately represents the experimentally obtained DADS(q)s.

Before performing structural refinement, we first evaluated how well the DFT-optimized structures, obtained using various computational methods, could describe the experimental curves without further refinement. As shown in Table S4, we explored several DFT functionals, including B3LYP-D3, CAM-B3LYP-D3, ωB97X, and PBE0-D3, to optimize the structural parameters for the GS, ^{3}(M/X)LCT, and ^{3}CC states. Among these functionals, the structure optimized with the PBE0-D3 functional provided the best agreement with the experimental data. However, as demonstrated in Figure S14b, noticeable discrepancies remained between the experimental and theoretical difference scattering curves, even with the PBE0-D3 structure. This result suggests that the DFT-optimized structures alone are insufficient to fully capture the structural dynamics in solution. Consequently, further refinement was necessary to better align the theoretical models with the experimental observations.

Starting from the PBE0-D3 structures, we attempted a refinement by allowing both Cu and I atom positions to vary. While this refinement improved the overall agreement between the theoretical and experimental curves, it also produced unrealistic interatomic distances, with the Cu–Cu distance reduced to 2.04 Å and the I–I distance to 3.68 Å—both significantly

shorter than the DFT-optimized values of 2.36 Å for Cu–Cu and 4.26 Å for I–I. These unrealistic distances likely resulted from overfitting, given the large number of adjustable parameters involved in the refinement process. To prevent overfitting, we adopted a more constrained refinement strategy. Specifically, we fixed the positions of the Cu atoms to those obtained from the DFT-optimized structures and refined only the positions of the iodine atoms. This strategy yielded good agreement with the experimental data, offering a reliable representation of the structural parameters while avoiding the introduction of unrealistic interatomic distances. As shown in Figure S14, the structure refinement significantly improved the fitting quality, demonstrating a close match between the theoretical and experimental curves. The structural parameters obtained from the refinement are summarized in Table S4.

The parameters used for the structural fitting for the ground state (GS) and two excited states of the $Cu_4I_4(py)_4$ cluster are shown in Figure S17. We employed two structural parameters for each structure to adjust the molecular structures anisotropically in two distinct dimensions: the xy-plane and the z-axis. In other words, we adjusted the structure so that it expands or contracts uniformly along the x-axis and y-axis, while expanding or contracting differently along the z-axis. Specifically, one parameter, denoted as f_{xy}, quantifies the overall expansion or contraction of the molecular structure within the xy-plane. The other parameter, denoted as f_z, manages the expansion or contraction along the z-axis. The arithmetic depiction of the positional changes for each atom, using the two scaling factors (f_{xy}, f_z) is as follows.

$$\begin{cases} x' = x \cdot f_{xy}\,(\%) \\ y' = y \cdot f_{xy}\,(\%) \\ z' = z \cdot f_{z}\,\,(\%) \end{cases} \tag{S20}$$

Here, x, y, and z represent the original coordinates of the I atoms in the core Cu_4I_4 structure of the cluster, whereas x', y', and z' denote the coordinates of the atoms after the structural modification. For GS, ^{3}CC, and ^{3}(M/X)LCT states, we employed two parameters for anisotropic structural adjustment because using a single parameter to uniformly expand or contract the structure in the x, y, and z directions did not satisfactorily fit the DADSs (fitting results not shown). This unsatisfactory fitting quality suggests that the DFT-optimized structure and the actual molecular structure in solution exhibit different symmetries: A simple expansion or contraction is inadequate to accurately represent the real molecular structure in solution.

Finally, the discrepancy between experimental and theoretical difference scattering curves was minimized by adjusting the structural parameters. To quantify the agreement between the calculated and experimental difference scattering curves, we calculated the reduced chi-square, χ_v^2, using the following equation:

$$\chi_v^2 = \frac{1}{N-p-1}\sum_i \sum_q \left(\frac{\mathrm{DADS}_i(q) - \mathrm{DADS}'_i(q)}{\sigma_i(q)} \right)^2 \tag{S21}$$

In Equation S21, $\mathrm{DADS}_i(q)$ and $\mathrm{DADS}'_i(q)$ stand for the experimentally measured and theoretically calculated i-th DADS, and σ_i is a standard error of the mean of $\mathrm{DADS}_i(q)$. Here, N and p refer to the number of data points and parameters, respectively. The q range of the scattering curves used for the fit was from 1.0 to 8.0 Å^{-1}. We note that the $\mathrm{DADS}_i(q)$ and $\mathrm{DADS}'_i(q)$ used for the fitting were PEPC-treated. The minimization of the χ_v^2 was performed using the MINUIT package from the CERN library, and the fit error was estimated by MINOS, a built-in algorithm in the MINUIT software.[11] During the structure refinement, the structural parameters were optimized by minimizing the χ_v^2. The fitting results are shown in Figure 3a, and the optimal parameters derived from the structure refinement are listed in Table S6. Table S7 lists the interatomic distances corresponding to the refined structures for the GS, ^{3}CC, and ^{3}(M/X)LCT states.

Fourier sine transform for converting *q*-space data into *r*-space information

To obtain a more intuitive real-space interpretation, we converted the experimentally and theoretically acquired $q\Delta S(q, t)$ curves to the radial distribution function, $r^2\Delta S(r, t)$ using the Fourier sine transform. The mathematical relation between $r^2\Delta S(r, t)$ and $q\Delta S(q, t)$ can be expressed as follows.

$$r^2 \Delta S(r,t) = \frac{r}{2\pi^2} \int_0^\infty q\Delta S(q,t) \sin(qr) e^{-q^2\alpha} dq \qquad \text{(S22)}$$

Here, α denotes a damping constant to account for the finite *q* range covered by the experiment. Figure S6 illustrates the $r^2\Delta S(r, t)$ obtained using Equation S22. Likewise, the PEPC-treated difference scattering curve, $q\Delta S^{\perp}(q, t)$, can also be transformed into $r^2\Delta S^{\perp}(r, t)$ using the Fourier sine transform provided below.

$$r^2 \Delta S^{\perp}(r,t) = \frac{r}{2\pi^2} \int_0^\infty q\Delta S^{\perp}(q,t) \sin(qr) e^{-q^2\alpha} dq \qquad \text{(S23)}$$

Here, the term "⊥" indicates that the curves are PEPC-treated. The resulting $q\Delta S^{\perp}(q, t)$ and the *r*-space representation of $q\Delta S^{\perp}(q, t)$ are depicted in Figure 2. Similarly, the same transformation can be applied to other various forms of difference scattering curves. The *r*-space representation of theoretical and experimental DADS can be obtained using Fourier sine transform to $\mathrm{DADS}_i(q)$ and $\mathrm{DADS}'_i(q)$. A mathematical representation of the Fourier sine transform is as follows:

$$\begin{aligned} r^2 \mathrm{DADS}_i(r) &= \frac{r}{2\pi^2} \int_0^\infty q\mathrm{DADS}_i(q) \sin(qr) e^{-q^2\alpha} dq \\ r^2 \mathrm{DADS'}_i(r) &= \frac{r}{2\pi^2} \int_0^\infty q\mathrm{DADS'}_i(q) \sin(qr) e^{-q^2\alpha} dq \end{aligned} \qquad \text{(S24)}$$

Here, $\mathrm{DADS}_i(r)$ and $\mathrm{DADS}'_i(r)$ refers to the *r*-space representation of the *i*-th experimental and theoretical DADS curve, respectively. The resulting $\mathrm{DADS}_i(r)$s are shown in Figures 3b and S16b, overlayed with $\mathrm{DADS}'_i(r)$s. For Equations S22, S23 and S24, we used $\alpha = 0.05$ Å^2 in our analysis. We note that, due to the presence of a beam block obstructing the direct X-ray beam, the low-*q* region ($q < 1.0$) of the experimental scattering curves is missing. However, excluding the low-*q* region during the Fourier transform can introduce undesired artifacts in their *r*-space representation. To suppress such artifacts, we complemented the low-*q* region of the experimental scattering curves with the theoretically calculated scattering curves. This procedure effectively eliminated the artifacts without significantly altering the shape of the *r*-space representation.[12]

Quantifying thermal dynamics in TRXL

Analyzing the TRXL data allows us to quantify the temperature change in the solution as a function of time delay. From the time-dependent temperature profile, we can estimate the amount of heat, Q(*t*), generated by the reaction. This heat is assumed to originate from two distinct sources: 1) photoexcited molecules that proceed through subsequent reaction cascades, populating long-lived excited states detectable in our experiment and releasing heat as the reaction progresses, and 2) photoexcited molecules that do not follow the reaction pathway (i.e., do not populate long-lived excited states) but instead relax directly back to the ground state, dissipating their excess energy as heat. By analyzing these contributions, we can determine the absolute concentration of the molecules in each category.

Based on this quantitative information, we can predict the portion of the initially excited population that rapidly relaxes back to the ground state. The specific number (42%) mentioned in the manuscript is derived from this analysis. To provide more context, we used the following equation, as established in previous studies:[4, 6-7]

$$Q(t) = Energy(t=0) - Energy(t)$$

$$= \frac{N_A}{R}\left[\sum_k (E_g + hv)c_k(0) - \sum_k E_k c_k(t)\right] + \frac{N_A}{R} f_{fast}\left[1 - \exp(-\frac{t}{k_{fast}})\right] \quad \text{(S25)}$$

Here, N_A is Avogadro's number, R is the ratio of the number of solvent molecules per solute, E_g (J·molecule^{-1}) is the absolute energy of the ground-state molecule, hv is the excitation energy, E_k (J·molecule^{-1}) is the energy of species k, f_{fast} denotes the fraction of rapidly relaxed species, and k_{fast} (s^{-1}) is the rate constant for the rapid relaxation, which occurs faster than the temporal resolution of TRXL (~100 ps).

Spectroscopic measurements

The absorption and emission spectra of $Cu_4I_4py_4$ were acquired using a UV-visible spectrometer (Shimadzu, UV2550) and a fluorometer (HORIBA FluoroMax PLUS), with the compound dissolved in acetonitrile at concentrations of 60 μM and 125 μM, respectively. The excitation wavelength for the emission spectrum measurement was set at 267 nm. Both absorbance and emission measurements were conducted using a quartz cell with a 1 cm thickness.

To further investigate the luminescence properties of the ^{3}(M/X)LCT state of $Cu_4I_4(py)_4$ in acetonitrile, time-correlated single-photon counting (TCSPC) measurements were conducted. The measurements were performed under two distinct environmental conditions: ambient air and inert (N_2-purged) environments. Acetonitrile was used as the solvent, and the concentration of $Cu_4I_4(py)_4$ was varied from 31.25 μM to 1 mM. The excitation wavelength for TCSPC measurements was set at 280 nm, corresponding to the same absorption band as the 267 nm wavelength used in the TRXL experiments. All measurements were conducted at room temperature (25°C) using a TCSPC instrument with a time resolution of <1.0 ns. The emission at 315 nm, corresponding to the high-energy band associated with the ^{3}(M/X)LCT state, was monitored throughout the experiments. The decay profiles obtained at various concentrations were analyzed using exponential fitting to extract the luminescence lifetimes. Further details regarding the experimental conditions, analysis, and insights gained are provided in the SI section titled "Understanding the variation in luminescence lifetimes of $Cu_4I_4(py)_4$: Solvent effects and concentration.".

Molecular dynamics (MD) simulation

We implemented MD simulations to describe the cage terms corresponding to the GS, ^{3}CC, and ^{3}(M/X)LCT states. All the simulations were performed by using the MOLDY 2.16e software[13] employing the Nose-Hoover thermostat[14-15] and periodic boundary condition. A solute molecule was embedded in 1024 acetonitrile solvent molecules in a virtual cubic cell with a size of 44.9577 Å. During the simulation, the structures of all molecules were kept fixed, and intermolecular interactions were estimated by considering Coulomb forces and Lennard-Jones potentials. The NBO atomic charges obtained from DFT calculations and universal force field (UFF) parameters were used to describe the intermolecular interactions.[16] All simulations were performed at an ambient temperature of 300 K with a solvent density of 0.786 g/cm^3. The system was equilibrated over 20 ps via coupling to a Nose-Hoover thermostat. The simulations were performed with a time step of 5 fs and the trajectories were followed up to 1 ns. The pair distribution functions (PDFs) were calculated from the simulated trajectories and used to calculate the scattering intensity of the solvent cages according to a well-established procedure.[4] The obtained cage term is incorporated into the analysis of the PEPC-treated TRXL data. Specifically, the cage term calculated

from the MD simulations, together with the solute term derived using the Debye equation, was employed to generate the theoretical solute-related terms, as described by the following equation:

$$\begin{aligned} S'_{\text{solute-related}}(q) &= S'_{\text{solute}}(q) + S'_{\text{cage}}(q) \\ \Delta S'_{\text{solute-related}}(q) &= \Delta S'_{\text{solute}}(q) + \Delta S'_{\text{cage}}(q) \end{aligned} \tag{S26}$$

where the prime symbol (′) indicates that the term is theoretically calculated rather than experimentally obtained. For each of the intermediates discussed in this work, ^{3}(M/X)LCT and ^{3}CC, the calculated terms $\Delta S'_{\text{solute-related}}(q)$, $\Delta S'_{\text{solute}}(q)$, and $\Delta S'_{\text{cage}}(q)$ are shown in Figure S18. For the analysis of PEPC-treated experimental data, as shown in Figure 2, and the raw experimental data, as shown in Figure S6, the two solute-related terms, $\Delta S'_{\text{solute-related}}(q)$s, calculated for ^{3}(M/X)LCT and ^{3}CC, along with two solvent heating components and a high-fluence artifact, were subsequently used as component for the LCF analysis.

After refining the structure using the solvent cage signal calculated from DFT-optimized structures, additional MD simulations were performed based on the refined molecular structures. The updated solvent cage term was then incorporated into the refinement process. This iterative approach was repeated until the refined structure achieved a satisfactory level of convergence, adhering to standard practices in such analyses.

Density functional theory (DFT) and time-dependent density functional theory (TD-DFT) calculation

We optimized the molecular structures corresponding to the GS, ^{3}CC, and ^{3}(M/X)LCT states, using DFT with four different functionals: B3LYP and PBE0 hybrid functionals[17-18], as well as CAM-B3LYP and ωB97X long-range-corrected functionals.[19-20] For the B3LYP, PBE0, and CAM-B3LYP functionals, Grimme’s D3 dispersion correction[21] was employed to better describe weak non-covalent interactions between pyridine rings. The geometry optimizations were performed without any symmetry constraints. We note that there were no imaginary frequencies for all optimized geometries. The key structural information of optimized structures, along with a comparison to experimental structures, is summarized in Table S4. Additionally, the energy gap between SOMOs of two triplet structures is represented in Figure S19.

To explore the nature of the excited states of $Cu_4I_4(py)_4$, TD-DFT calculations were performed. For a better description of the charge transfer character within the molecule, CAM-B3LYP with D3 dispersion corrections and the LC-ωHPBE long-range-corrected functional were employed for the TD-DFT calculations to address inaccuracies in describing charge transfer character typically encountered when using traditional functionals such as B3LYP or PBE0. The absorption spectra of $Cu_4I_4(py)_4$ were calculated with 50 low-lying singlet states for both functionals. To match the experimental absorption spectra, Lorentzian broadening with 0.1 eV FWHM was employed for the calculated spectra (see Figure S20). The difference between the calculated vertical excitation energy for the ^{1}CC state and that for the ^{1}(M/X)LCT state was below 0.3 eV for both CAM-B3LYP and LC-ωHPBE functionals, confirming the proximity of the two different electronic states. For the transition density matrix (TDM) analysis, the results of TD-DFT calculation with the LC-ωHPBE functional and the CAM-B3LYP functional were employed.

For a comprehensive analysis of the charge transfer character of the photoexcited molecule, TDM analysis was performed. First, the molecule was divided into three distinct fragments corresponding to copper, iodine and pyridines. Using the one-electron transition density matrix (1-TDM) calculated with TD-DFT, the Ω-matrix was obtained, which represents the charge transfer character between the three fragments. The results of TDM analysis are shown in Figure S3. This analysis not only validates the contributions of two predominant types of charge transfer (CC and (M/X)LCT) upon excitation at 267 nm, but also provides detailed values quantifying the charge transfer character between fragments. The populations derived from TDM analysis are quantitative. Still, the results are highly dependent on the choice of functional and the calculation method of

atomic integrals required for TDM analysis.[22] For instance, a comparison of the results obtained using the LC-ωhPBE functional (shown in Figure S3) and the CAM-B3LYP functional (shown in Figure S4) reveals substantial differences. In particular, the CAM-B3LYP results show distinct deviations in the LLCT character contributions for the (M/X)LCT states. Specifically, the LLCT character population is markedly smaller for the S_{15} and S_{16} states calculated with the CAM-B3LYP functional compared to the S_{12} and S_{13} states predicted by the LC-ωhPBE functional (with the S_{15} and S_{16} states from CAM-B3LYP corresponding to the S_{12} and S_{13} states from LC-ωhPBE, respectively). Despite these differences, the CC and (M/X)LCT characteristics of the excited states remain consistent across both functionals. For this reason, our characterization of the excited states focuses on these key features—namely, the CC and (M/X)LCT characters—as they provide a reliable basis for understanding the nature of the photoinduced dynamics in $Cu_4I_4(py)_4$.

For all calculations, the def2-TZVPP (def2-SVP for C, N, H atoms) basis set was employed.[23] To account for the effect of the solvent polarity (acetonitrile), a conductor-like polarizable continuum model (C-PCM) was used.[24] DFT and TD-DFT calculations were conducted with Gaussian 16 software[25] and TDM analysis was performed with the Theodore 3.0 package.[26]

Supporting Discussions

Characterization of the two emissive states in $Cu_4I_4(py)_4$

Numerous experiments and theoretical investigations have explored the two emissive states of $Cu_4I_4(py)_4$.[27-35] However, the nomenclature for these emissive states has evolved over time. These states were categorized as HE (Higher Energy) and LE (Lower Energy) states based on their respective energy levels. Initial studies, integrating absorption and emission spectra with ab initio calculations, attributed the HE state to possess MLCT character, whereas the LE state exhibited MCC (Metal Cluster-Centered) character.[29, 34] Later, employing more detailed approaches, the authors further identified XMCT character in the LE state, in addition to the MCC character, leading to the use of the term "CC character" to describe the combination of both characteristics.[27, 30-31, 35] Likewise, their calculations revealed that the HE state predominantly exhibits XLCT character rather than MLCT character.[27, 30-31, 35] These findings have long been accepted, and currently, the two emissive states, HE and LE, are now commonly assigned as XLCT and CC states, respectively.[28, 32]

Contrary to previous assignments, transition matrix analysis presented in Figure S3 reveals that transitions previously categorized as the XLCT state primarily exhibit MLCT character, although the XLCT character also makes a substantial contribution and should not be overlooked. Accordingly, we assigned this energy state as having a (M/X)LCT mixed character. The transitions corresponding to the CC state exhibit MCC, XMCT, and notably, in contrast to the previous assignments, also exhibit MXCT (Metal-to-halide charge transfer) character. Importantly, the contribution of the MXCT character even exceeds that of the XMCT character. We suggest that the discrepancy in energy state assignment arises from the differing methodologies employed across studies. While previous assignments were based on qualitative visualizations of molecular orbitals, our approach utilizes transition matrix analysis, which quantifies the contributions of each charge transfer component, thus allowing for a more accurate and detailed characterization of the energy states.

Investigation of the potential for long-lived photochemical products that do not decay within the observed time window

Examining the RSV at late time delays (> 500 ns) revealed that the RSV values do not converge to zero. This feature results in unsatisfactory exponential fitting quality unless a constant term is included, particularly for the RSV2. Consequently, we included a constant term in the fitting process. For the RSV1, the constant term was fixed to zero. The presence of this constant term suggests the potential existence of a long-lived species that persists beyond the observed time window. To explore this possibility, we applied a modified kinetic model that accounts for the formation of such a long-lived species.

In this modified model, we hypothesized the existence of a long-lived photochemical product and attempted to extract the DADS associated with the species by calculating the third DADS ($DADS_3$). The results of this analysis, shown in Figure S10, indicate that the $DADS_3$ curve contains only noise and lacks any meaningful structural features. This strongly suggests that either no long-lived photochemical products contribute to the observed scattering signal, or, if such products are generated, their quantities are below the detection limit of our measurements. These findings confirm that the ^{3}CC state fully decays to the ground state with the observed time constant of 202 ns. The non-zero RSV values observed at late time delays are therefore attributed to experimental artifacts and noise, rather than any structural signal from long-lived species.

Understanding the variation in luminescence lifetimes of $Cu_4I_4(py)_4$: Solvent effects and concentration

The lifetimes of the ^{3}(M/X)LCT and ^{3}CC states observed in our experiment—1.21 ns and 202 ns, respectively—differ significantly from the previously reported values of 450 ns and 10.6 μs in toluene. This discrepancy can be attributed to the following three key factors: solvent effects, solvent coordination, and solute concentration. First, solvent dependency plays a crucial role in determining emission lifetimes. Our experiment was conducted in acetonitrile, a solvent with properties markedly different from toluene, which was used in the previous studies. As reported in the previous studies, the emission lifetimes of $Cu_4I_4(py)_4$ clusters vary considerably depending on the solvent environment.[27-28] For example, while the lifetime of the ^{3}(M/X)LCT state is 450 ns in toluene, it decreases to 76 ns in dichloromethane (CH_2Cl_2). This trend suggests that acetonitrile, for which no lifetimes have previously been reported, likely contributes to the shorter lifetimes observed in our study. Second, the coordinating nature of acetonitrile may promote luminescence quenching, particularly in the ^{3}CC state. Another previous study reported a significant reduction in emission in coordinating solvents such as CH_2Cl_2 and acetone. In acetonitrile, which exhibits an even stronger coordinating character, the emission signal was reported to be nearly undetectable.[29] Consistent with these findings, we also observed extremely weak luminescence signals for $Cu_4I_4(py)_4$ in acetonitrile. Although earlier studies, conducted approximately 30 years ago, found the emission in acetonitrile too weak to measure accurately, advances in modern equipment sensitivity enabled us to capture these weak emissions. Lastly, the higher solute concentration used in our experiment (5 mM) compared to previous experiments (0.05–1.35 mM) may have further contributed to the shorter lifetimes through self-quenching. At higher concentrations, bimolecular quenching becomes more pronounced, shortening the emission lifetimes of the ^{3}(M/X)LCT state.[33] Similar concentration-dependent quenching effect was also observed in another study.[27] Taken together, these three factors—solvent dependency, coordination-induced quenching, and self-quenching at higher concentrations—explain the differences between the lifetimes observed in our acetonitrile experiment and those reported for toluene.

To further investigate these factors and gain deeper insight into the luminescence dynamics of $Cu_4I_4(py)_4$, we conducted TCSPC measurements under controlled conditions. These experiments aimed to examine how increasing concentration of $Cu_4I_4(py)_4$ influences luminescence decay and to develop a kinetic model for the process. Measurements were conducted in two different environments: ambient air and an inert (N_2-purged) atmosphere. To maintain consistency with the TRXL experiment, acetonitrile, the same solvent used in the TRXL experiments, was used for these measurements. The results, summarized in Table S5 and plotted in Figure S12, demonstrate a concentration dependence of the luminescence lifetime. At

the lowest concentration (31.25 μM) and under inert conditions, the lifetime was observed to be 3.81 ns. As the concentration increased, the lifetime decreased significantly, reaching 2.55 ns at 1 mM. This concentration-dependent lifetime indicates the presence of a pathway whose rate is influenced by the concentration of the $Cu_4I_4(py)_4$ species, further suggesting that a self-quenching bimolecular interaction plays a significant role in this pathway. Nevertheless, Figure S12, which plots the inverse of the luminescence lifetime as a function of concentration, indicates that the rate of the decay process does not converge to zero at near-zero concentrations. This suggests the existence of decay pathways that do not involve interactions between $Cu_4I_4(py)_4$ molecules.

A comparison of results from measurements conducted under ambient air and inert conditions revealed two distinct concentration-independent decay processes, where the rate is unaffected by the concentration of $Cu_4I_4(py)_4$. The decay rate in ambient air was significantly faster than that observed in inert conditions. At very low concentrations, as indicated by the y-intercept in Figure S12c, the decay rate in ambient air was approximately twice as fast as in the inert environment. This observation suggests the existence of two independent pathways contributing to the $Cu_4I_4(py)_4$-concentration-independent decay process of the ^{3}(M/X)LCT state. One pathway is likely associated with the quenching of the triplet excited state by oxygen, while the other operates independently of oxygen and corresponds to the radiative decay process of the ^{3}(M/X)LCT state.

To interpret these results, we propose a kinetic model that includes both $Cu_4I_4(py)_4$-concentration-independent and $Cu_4I_4(py)_4$-concentration-dependent processes. At low concentrations, the decay is dominated by the following two processes, which can be described as follows.

1. Oxygen-involved process:

$$^3\text{(M/X)LCT} + \text{O}_2 \xrightarrow{k_{\text{O}_2}} \text{GS} \quad \text{(S27)}$$

2. Oxygen-independent process:

$$^3\text{(M/X)LCT} \xrightarrow{k} \text{GS} \quad \text{(S28)}$$

Here, k_{O2} represents a second-order reaction rate constant for the quenching effect of oxygen and k denotes the rate constant for the oxygen-independent process. Since the focus of this study is not on distinguishing these two processes, for simplicity, we represent the combined contribution of these pathways with a single rate constant, k_{decay}. This effective rate constant is defined as:

$$k_{decay} = k_{\text{O}_2} \cdot [\text{O}_2] + k \quad \text{(S29)}$$

At higher concentrations, a self-quenching bimolecular interaction becomes significant, which can be represented as follows.

$$^3\text{(M/X)LCT} + \text{GS} \xrightarrow{k_{bi}} \text{GS} + {}^3\text{CC} \quad \text{(S30)}$$

Here, k_{bi} is a second-order reaction rate constant for the bimolecular process between $Cu_4I_4(py)_4$ molecules. The total decay rate (k_{total}) can be expressed as:

$$k_{total} = k_{\text{O}_2} \cdot [\text{O}_2] + k + k_{bi} \cdot [\text{GS}] = k_{decay} + k_{bi} \cdot [\text{GS}] \quad \text{(S31)}$$

The observed lifetimes and their dependence on concentration and oxygen conditions support this kinetic model. Analysis of the TCSPC data we measured yields the values of k_{decay} and k_{bi} as 2.514×10^8 s^{-1} and 1.432×10^{11} M$^{-1}\cdot$s^{-1}, respectively. Based on k_{decay} and k_{bi}, the calculated apparent time constant for the decay of ^{3}(M/X)LCT under the experimental conditions used in the TRXL experiment (5 mM of $Cu_4I_4(py)_4$) is 1.03 ns which closely aligns with the time constant determined from our TRXL experiments (1.21 ± 0.61 ns). The corresponding kinetic model is illustrated in Figure S12d. Using the values of k_{bi} and k_{decay}, we can estimate the rates of the ^{3}(M/X)LCT → ^{3}CC transition and the ^{3}(M/X)LCT → GS transition at a 5 mM concentration,

which corresponds to the conditions used in the TRXL experiment. At the $Cu_4I_4(py)_4$ concentration of 5 mM, the pseudo-first-order rate constant for the ^{3}(M/X)LCT → ^{3}CC transition is approximately 7.16×10^8 s^{-1}, calculated by multiplying k_{bi} by the concentration of $Cu_4I_4(py)_4$. Comparing this value with k_{decay}, the ratio of the calculated rate constants for the ^{3}(M/X)LCT → ^{3}CC transition and the ^{3}(M/X)LCT → GS transition is approximately 74:26. By applying this ratio to the apparent rate constant obtained from the TRXL experiment (8.26×10^8 s^{-1}, corresponding to (1.21 ns)$^{-1}$), the pseudo-first-order rate constant for the ^{3}(M/X)LCT → ^{3}CC transition is determined to be 6.13×10^8 s^{-1} (74% of the apparent rate constant). Considering the 5 mM concentration of $Cu_4I_4(py)_4$, the corresponding bimolecular reaction rate constant is calculated as 1.23×10^{11} M^{-1}·s^{-1}, obtained by dividing the pseudo-first-order rate constant (6.13×10^8 s^{-1}) by the concentration (5 mM). The rate constant for the ^{3}(M/X)LCT → GS transition is calculated as 2.15×10^8 s^{-1} (26% of the apparent rate constant), which corresponds to a lifetime of 4.65 ns.

Supporting Tables

Table S1. Excitation wavelength and oscillator strength of the first five lowest excited states with significant oscillator strengths (> 0.05).

$S_0 \rightarrow S_n$ transition	Oscillator strength	Excitation wavelength (nm / eV)
S_7	0.166	258.4 / 4.80
S_8	0.140	258.2 / 4.80
S_9	0.140	258.2 / 4.80
S_{12}	0.060	253.5 / 4.89
S_{13}	0.060	253.5 / 4.89

Table S2. Hole-electron distribution in $Cu_4I_4(py)_4$. To examine the charge transfer characteristics of the excited states of $Cu_4I_4(py)_4$ quantitatively, we categorized the hole and electron distributions into two segments based on their locations: (i) the copper and iodine frame, referred to as "cubane", and (ii) the pyridine ligands. We then quantified the amounts of holes and electrons corresponding to each segment by analyzing the Ω-matrix derived from TD-DFT calculations.

h^+ / e^-	cubane h^+	cubane e^-	pyridine h^+	pyridine e^-
S7	0.993	0.991	0.008	0.010
S8	0.966	0.923	0.035	0.078
S9	0.966	0.923	0.035	0.078
S12	0.722	0.158	0.278	0.842
S13	0.722	0.158	0.278	0.842

Table S3. Hole-electron distribution in $Cu_4I_4(py)_4$. A similar analysis to that shown in Table S2 was conducted; however, here, the “cubane” category from Table S2 is further divided into more detailed classifications corresponding to “Cu” and “I” for a more detailed examination.

h^+ / e^-	Cu h^+	Cu e^-	I h^+	I e^-	pyridine h^+	pyridine e^-
S_7	0.697	0.523	0.295	0.468	0.008	0.010
S_8	0.710	0.487	0.256	0.436	0.035	0.078
S_9	0.710	0.487	0.256	0.436	0.035	0.078
S_{12}	0.565	0.093	0.157	0.065	0.278	0.842
S_{13}	0.565	0.093	0.157	0.065	0.278	0.842

Table S4. Structural parameters for the GS, ^{3}(M/X)LCT, and ^{3}CC states, obtained through structure refinement (labeled as "Experiment") and quantum chemical calculations.

		Experiment	B3LYP-D3	CAM-B3LYP-D3	ωb97X	PBE0-D3
Fit quality (χ_v^2)		4.79	11.03	7.99	9.41	6.45
GS	Cu–Cu (Å)	2.642–2.679	2.676–2.694	2.683–2.696	2.710–2.712	2.642–2.679
	I–I (Å)	4.401–4.525	4.633–4.638	4.558–4.559	4.577–4.586	4.526–4.571
	Cu–I (Å)	2.601–2.701	2.684–2.855	2.668–2.780	2.695–2.789	2.678–2.737
	Cu–N-C (°)	118.7–122.5	119.1–122.0	118.9–122.3	119.6–121.8	118.7–122.5
	N–Cu–I (°)	103.1–109.8	98.7–110.5	101.5–110.4	101.8–109.0	103.1–109.8
	Cu–I-Cu (°)	59.6–59.9	56.9–58.9	58.5–59.9	58.7–59.8	58.4–58.8
^{3}(M/X)LCT	Cu–Cu (Å)	2.663–2.800	2.690–2.909	2.692–2.952	2.706–2.889	2.663–2.800
	I–I (Å)	4.253–4.434	4.434–4.636	4.354–4.585	4.408–4.594	4.403–4.549
	Cu–I (Å)	2.591–2.671	2.684–2.770	2.656–2.750	2.676–2.756	2.657–2.720
	Cu–N-C (°)	118.7–123.3	118.6–122.8	118.8–122.4	119.2–122.8	118.7–123.3
	N–Cu–I (°)	104.9–114.5	103.2–115.4	104.4–111.5	104.3–115.3	103–108.9
	Cu–I-Cu (°)	60.1–64.5	58.1–64.5	58.6–66.2	59.0–64.4	58.7–64.5
^{3}CC	Cu–Cu (Å)	2.358–3.365	2.367–3.450	2.313–3.512	2.519–2.672	2.358–3.365
	I–I (Å)	4.196–5.880	4.339–6.392	4.237–6.300	4.396–6.718	4.256–6.229
	Cu–I (Å)	2.503–4.647	2.664–4.931	2.620–4.974	2.576–4.965	2.628–4.824
	Cu–N-C (°)	119.7–121.1	119.8–120.9	120.3–120.7	118.1–123.3	119.7–121.1
	N–Cu–I (°)	87.2–120.1	87.2–118.1	86.8–125.1	86.0–125.3	87.1–118.9
	Cu–I-Cu (°)	43.7–62.7	39.5–58.8	40.2–61.6	32.7–58.8	40.4–51.3

*All basis sets used in the quantum calculations are as follows: def2-TZVPP for most atoms and def2-SVP for N, C, and H.

**The structures labeled as "Experiment" were determined through the structure refinement process, as detailed in the "Structure refinement by structural fitting analysis" section of SI.

Table S5. Time constants obtained from TCSPC measurements of $Cu_4I_4(py)_4$ in acetonitrile at various concentrations under ambient conditions and with N_2-purging. The measurements were conducted at the emission wavelength of the ^{3}(M/X)LCT state (315 nm), with an excitation wavelength of 280 nm.

Ambient condition		N_2–purged condition	
Conc	τ_1 / ns	Conc	τ_1 / ns
31.25 μM	3.81	31.25 μM	6.69
125 μM	3.73	125 μM	6.09
250 μM	3.68	250 μM	5.01
500 μM	2.98	500 μM	4.83
1 mM	2.55	1 mM	3.33

Table S6. Structural parameters for the GS, ^{3}(M/X)LCT, and ^{3}CC states optimized through the structure refinement. The interatomic pair distances listed in this table are averaged values of multiple distances. The detailed information on all interatomic pair distances is listed in Table S7.

		GS	^{3}(M/X)LCT	^{3}CC
f_{xy}		99.0 ± 0.2 %	96.6 ± 0.6 %	98.6 ± 0.2 %
f_{z}		95.3 ± 0.4 %	98.3 ± 1.1 %	91.3 ± 0.2 %
Cu–Cu	xy plane	2.679 Å	2.734 Å	2.358 Å
	z axis (short)	2.643 Å	2.664 Å	2.507 Å
	z axis (long)	2.643 Å	2.791 Å	3.117 Å
I–I[b]	xy plane	4.525 ± 0.010 Å	4.307 ± 0.026 Å	4.196 ± 0.004 Å
	z axis (short)	4.401 ± 0.020 Å	4.320 ± 0.047 Å	4.561 ± 0.010 Å
	z axis (long)	4.401 ± 0.020 Å	4.421 ± 0.048 Å	5.880 ± 0.012 Å
Cu–I[b]	xy plane	2.695 ± 0.006 Å	2.622 ± 0.016 Å	2.618 ± 0.005 Å
	z axis (short)	2.601 ± 0.012 Å	2.631 ± 0.029 Å	3.630 ± 0.007 Å
	z axis (long)[a]	2.601 ± 0.012 Å	2.666 ± 0.029 Å	3.667 ± 0.007 Å

[a]Only the adjacent Cu and I atoms are considered.

[b]Errors are estimated from the errors in f_{xy} and f_{z}, which were determined through structure refinement analysis.

Table S7. All interatomic distances in the Cu_4I_4 core of the GS, ^{3}(M/X)LCT, and ^{3}CC states, optimized through structure refinement.

		GS	^{3}(M/X)LCT	^{3}CC
f_{xy}		99.0 ± 0.2 %	96.6 ± 0.6 %	98.6 ± 0.2 %
f_z		95.3 ± 0.4 %	98.3 ± 1.1 %	91.3 ± 0.2 %
Cu–Cu	xy plane	2.679 Å	2.668 Å	2.358 Å
		2.679 Å	2.800 Å	2.358 Å
	z axis	2.643 Å	2.663 Å	2.507 Å
		2.643 Å	2.665 Å	2.507 Å
		2.643 Å	2.788 Å	2.869 Å
		2.643 Å	2.793 Å	3.365 Å
I–I	xy plane	4.525 ± 0.009 Å	4.253 ± 0.025 Å	4.196 ± 0.008 Å
		4.525 ± 0.009 Å	4.361 ± 0.026 Å	4.196 ± 0.008 Å
	z axis	4.401 ± 0.017 Å	4.300 ± 0.047 Å	4.363 ± 0.010 Å
		4.401 ± 0.017 Å	4.339 ± 0.047 Å	4.758 ± 0.010 Å
		4.401 ± 0.017 Å	4.407 ± 0.048 Å	5.880 ± 0.012 Å
		4.401 ± 0.017 Å	4.435 ± 0.048 Å	5.880 ± 0.012 Å
Cu–I	xy plane	2.689 ± 0.005 Å	2.591 ± 0.015 Å	2.503 ± 0.005 Å
		2.689 ± 0.005 Å	2.607 ± 0.016 Å	2.503 ± 0.005 Å
		2.689 ± 0.005 Å	2.612 ± 0.016 Å	2.529 ± 0.005 Å
		2.689 ± 0.005 Å	2.614 ± 0.016 Å	2.529 ± 0.005 Å
		2.701 ± 0.005 Å	2.626 ± 0.016 Å	2.687 ± 0.005 Å
		2.701 ± 0.005 Å	2.633 ± 0.016 Å	2.687 ± 0.005 Å
		2.701 ± 0.005 Å	2.646 ± 0.016 Å	2.753 ± 0.006 Å
		2.701 ± 0.005 Å	2.648 ± 0.016 Å	2.753 ± 0.006 Å
	z axis	2.601 ± 0.010 Å	2.629 ± 0.029 Å	3.630 ± 0.007 Å
		2.601 ± 0.010 Å	2.632 ± 0.029 Å	3.630 ± 0.007 Å
		2.601 ± 0.010 Å	2.661 ± 0.029 Å	3.667 ± 0.007 Å
		2.601 ± 0.010 Å	2.672 ± 0.029 Å	3.667 ± 0.007 Å
		4.346 ± 0.017 Å	4.294 ± 0.047 Å	4.357 ± 0.008 Å
		4.346 ± 0.017 Å	4.298 ± 0.047 Å	4.357 ± 0.008 Å
		4.346 ± 0.017 Å	4.298 ± 0.047 Å	4.647 ± 0.009 Å
		4.346 ± 0.017 Å	4.449 ± 0.047 Å	4.647 ± 0.009 Å

Supporting Figures

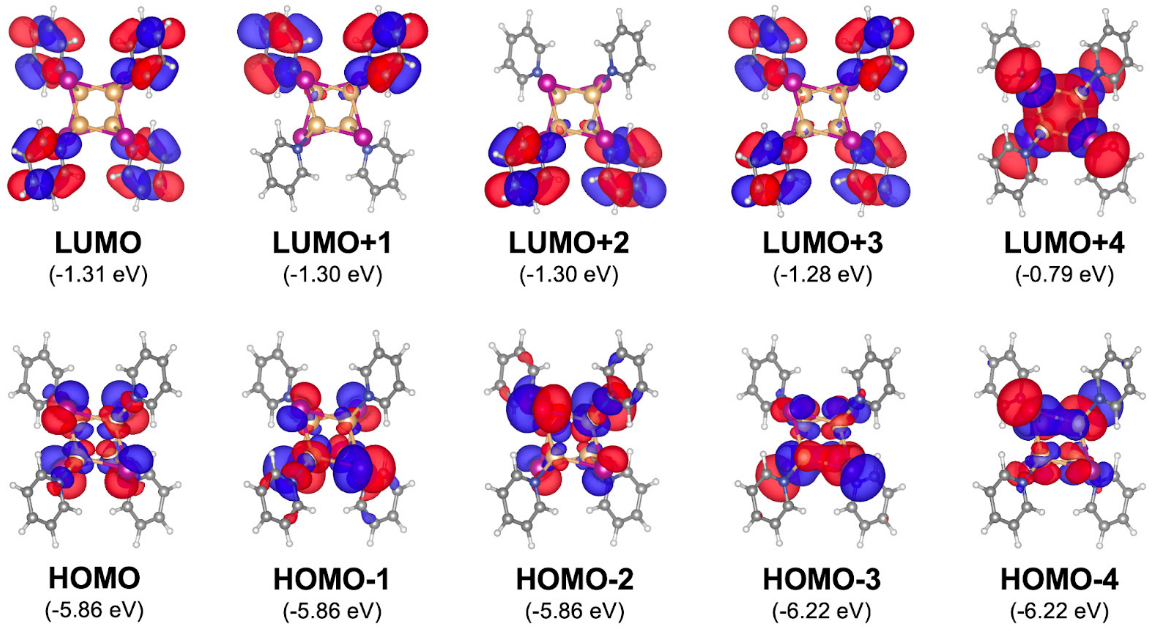

Figure S1. Molecular orbital diagrams of the ground state $Cu_4I_4(py)_4$. The orbital energies were obtained using DFT calculations with PBE0-D3/def2-TZVPP (def2-SVP). The five orbitals from HOMO-4 to HOMO each exhibit mixed characters of the 4p orbital of I and the 3d orbital of Cu. The orbitals from LUMO to LUMO+3 predominantly show π orbital characters of the py ligands. LUMO+4 displays mixed characters of 5s orbital of Cu and 4p orbital of I.

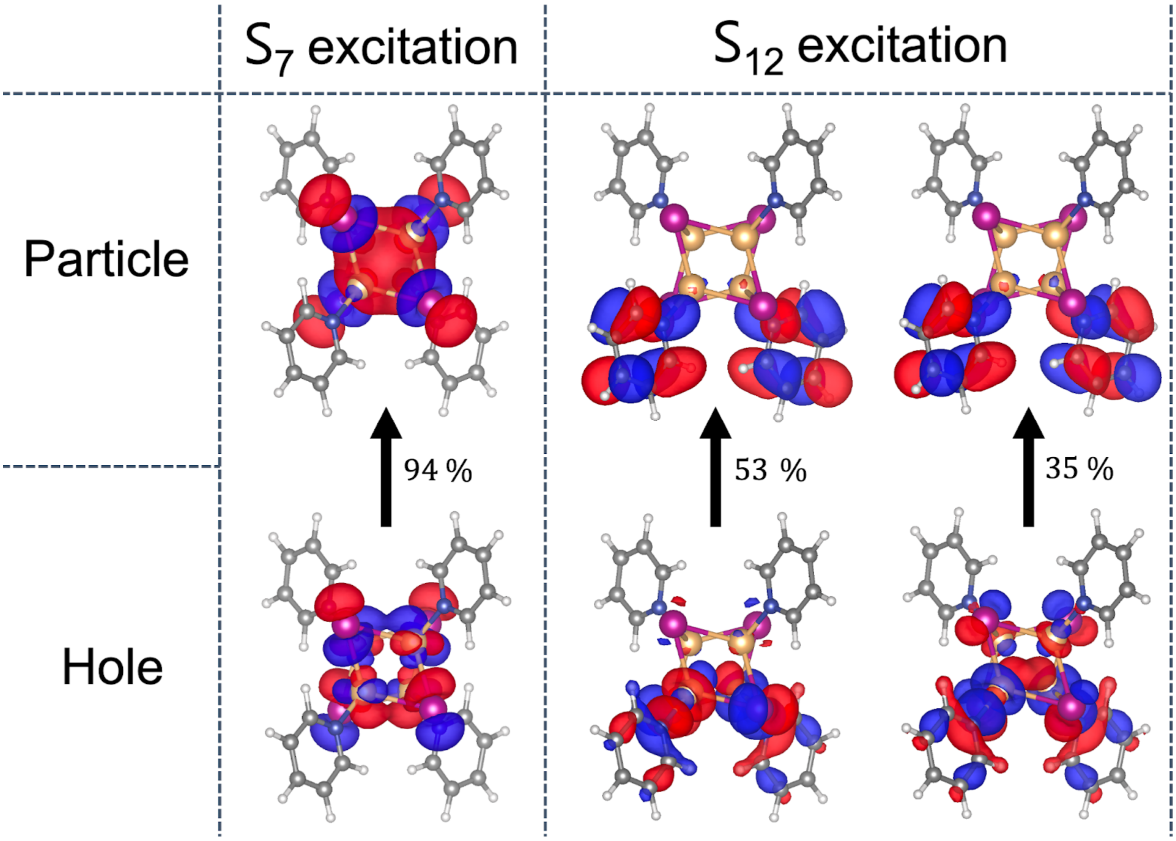

Figure S2. Natural transition orbitals (NTOs) for S_0 → S_7 and S_0 → S_{12} transitions. S_0 → S_7 (left) is a representative transition exhibiting CC character, while S_0 → S_{12} (right) is a representative transition displaying (M/X)LCT character. For each electronic transition, the major contributing sub-transitions are illustrated, along with their quantitative contributions.

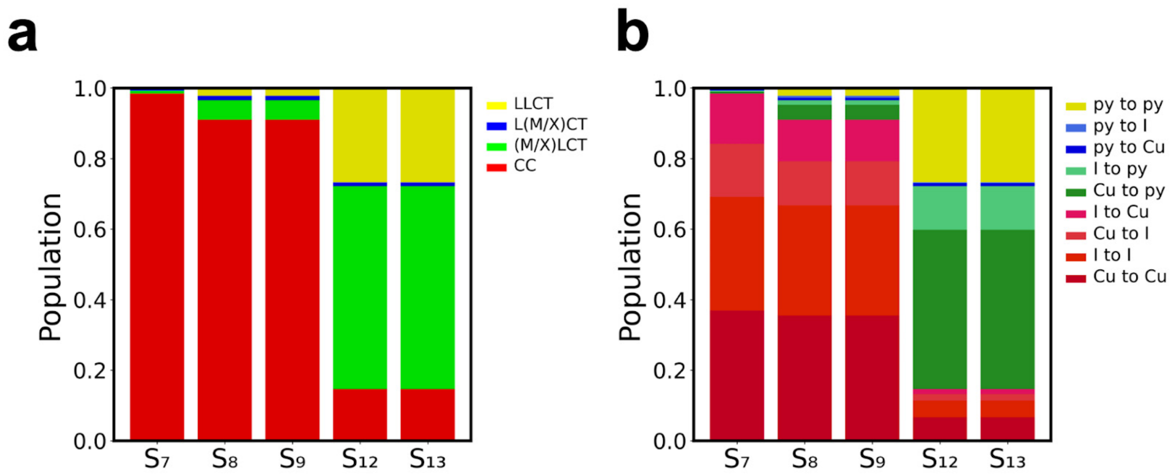

Figure S3. Charge transfer characters of excited states derived from transition density matrix analysis using the LC-ωhPBE functional. Here, the populations of charge transfer characters for the first five lowest excited states with significant oscillator strengths (> 0.05) in the $Cu_4I_4(py)_4$ molecule are shown. We analyzed the charge transfer characters by fragmenting the molecule in two ways: (a) into two fragments (cubane and pyridine) and (b) into three fragments (copper, iodine, and pyridine), quantitatively assessing the charge transfer characters between these fragments. In panel (a), the populations of CC transitions are shown in red, (M/X)LCT transitions in green, L(M/X)CT transitions in blue, and LLCT transitions in yellow. The color scheme in (a) is consistently applied in (b), those exhibiting the CC character are colored in shades of red, those exhibiting (M/X)LCT character in shades of green, those exhibiting L(M/X)CT character in shades of blue, and those exhibiting LLCT character in shades of yellow.

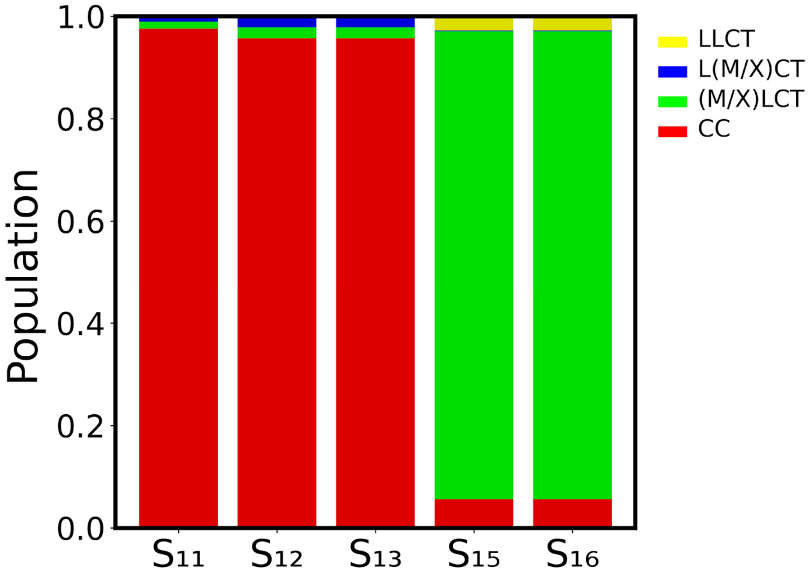

Figure S4. Transition density matrix analysis calculated using the CAM-B3LYP functional. Here, the populations of charge transfer characters for the first five lowest excited states with oscillator strengths larger than 0.05 in the $Cu_4I_4(py)_4$ molecule are presented. To analyze the charge transfer characters, we fragmented the molecule into three fragments (copper, iodine, and pyridine) and quantitatively assessed the charge transfer between these fragments. The populations of CC transitions are shown in red, (M/X)LCT transitions in green, L(M/X)CT transitions in blue, and LLCT transitions in yellow. The S_{11}, S_{12}, and S_{13} states, corresponding to the S_7, S_8, and S_9 states calculated with LC-ωhPBE, exhibit a predominant CC character. Meanwhile, the S_{15} and S_{16} states obtained with CAM-B3LYP primarily exhibit (M/X)LCT character and align with the S_{12} and S_{13} states predicted by LC-ωhPBE, which also mainly show (M/X)LCT character.

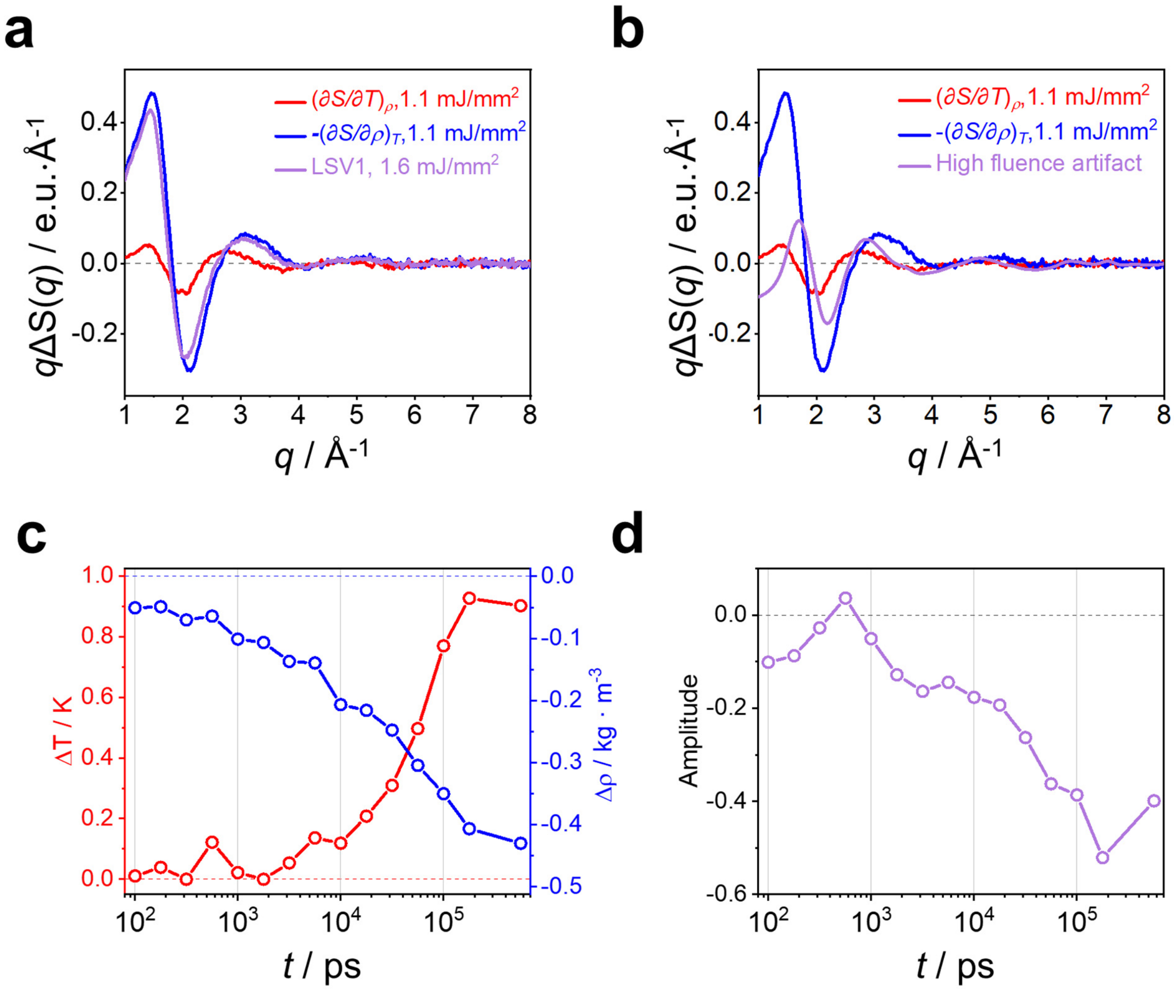

Figure S5. Solvent heating signals measured under two different laser fluence conditions, the retrieved high-laser-fluence-associated artifact, and the time-dependent changes in Δ*T*, Δ*ρ*, and the artifact contribution. (a) Comparison of the $(\partial S/\partial T)_\rho$ (red curve) and $(\partial S/\partial \rho)_T$ (blue curve) terms obtained with the 1.1 mJ/mm^2 fluence and the first left singular vector derived from SVD analysis of the solvent heating signal measured with the 1.6 mJ/mm^2 fluence (purple curve). (b) Comparison of the artifact component associated with high laser fluence (purple curve), alongside the $(\partial S/\partial T)_\rho$ (red curve) and $(\partial S/\partial \rho)_T$ (blue curve) terms obtained with the 1.1 mJ/mm^2 fluence, represented in *q*-space. (c) Time-dependent changes in the temperature and density of the solvent. (d) Time-dependent contribution of the artifact component. All profiles in (c) and (d) were obtained by performing a linear combination fitting of ΔS(*q*, *t*) using the three components shown in (b) and the theoretical difference scattering curves corresponding to the ^{3}(M/X)LCT and ^{3}CC states depicted in Figure S18, for each time delay.

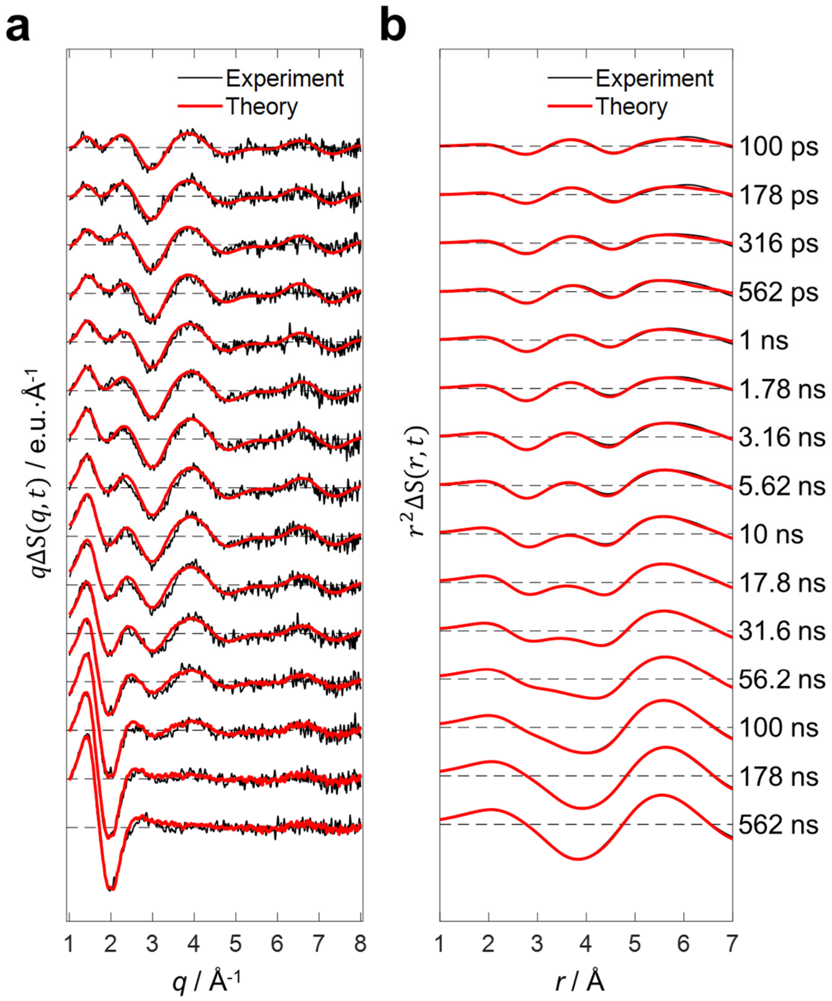

Figure S6. Experimental TRXL data and theoretical fits for $Cu_4I_4(py)_4$ in acetonitrile with 267 nm excitation. (a) Experimental difference scattering curves (black) with corresponding simulated theoretical fits (red). Theoretical fits were derived through linear combination fitting (LCF) of $\Delta S(q, t)$ for each time delay. (b) Difference radial distribution function, $r^2\Delta S(r, t)$, obtained through the Fourier sine transformation of $q\Delta S(q, t)$ shown in (a).

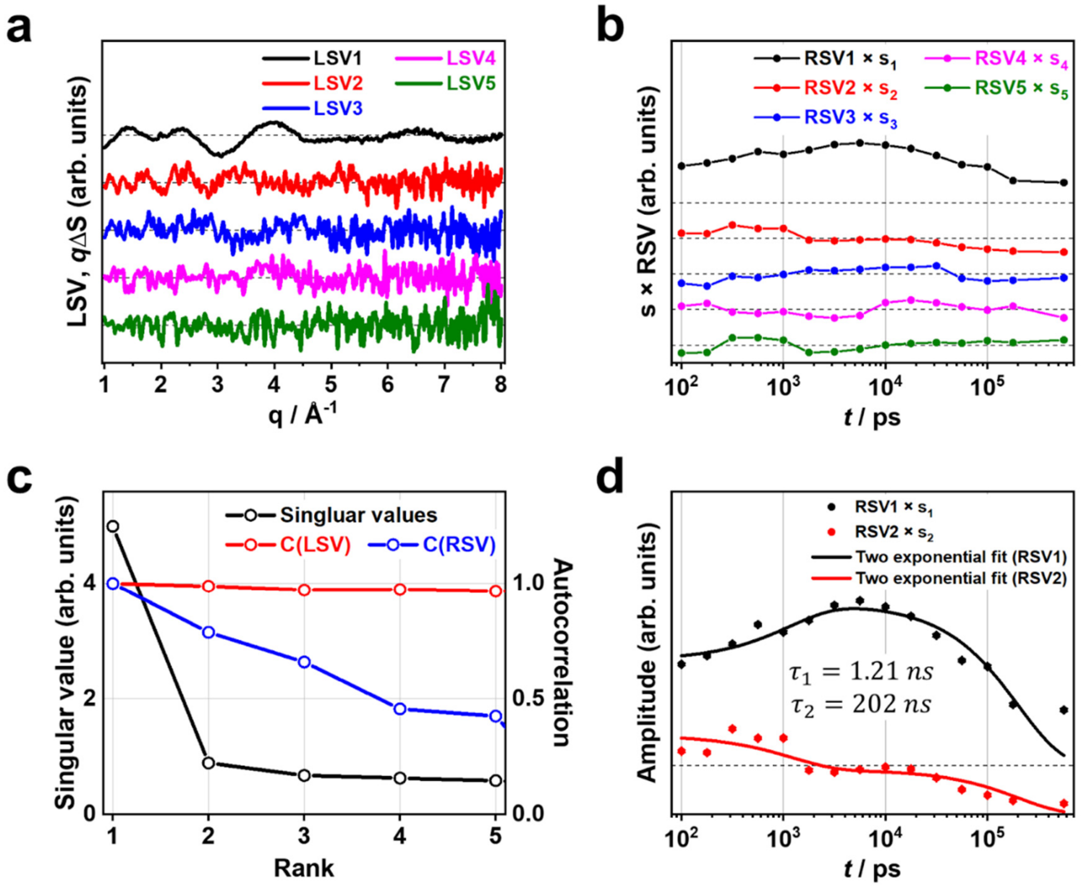

Figure S7. SVD analysis of the PEPC-treated TRXL data of [$Cu_4I_4(py)_4$]. (a, b) The first five left (a) and right (b) singular vectors (LSVs and RSVs) of the TRXL data. The RSVs are weighted by the corresponding singular values. (c) The singular values and the autocorrelation values, noted as C, for RSVs and LSVs. The singular values and autocorrelation values, together with the overall features of the LSVs and RSVs, indicate that two signal components contribute significantly to the TRXL data. (d) The fit (solid lines) of the first two RSVs (circles) with a sum of a constant and two exponential functions sharing the time constants. For the RSV1, the constant term was fixed to zero. The time constants obtained from the fitting are 1.21 ± 0.61 ns and 202 ± 37 ns, respectively.

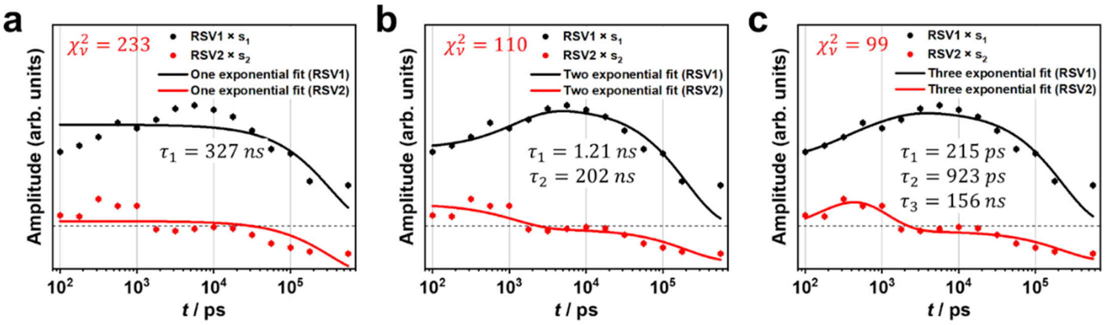

Figure S8. Exponential fitting analysis of the first two RSVs with changing the number of exponential functions. (a–c) Fits of the RSV with (a) one exponential, (b) two exponentials, and (c) three exponentials, each including a constant term. For RSV1, the constant term was constrained to zero during the fitting, while for RSV2, the constant term was included as a free parameter. In the top-left corner of each figure, the fit quality is indicated by the reduced chi-square value, χ_v^2, providing an assessment of the goodness of fit. Using a single exponential, as illustrated in case (a), falls short in accurately describing the observed time-dependent changes in the signal. Employing three exponentials, as demonstrated in case (c), does not exhibit a significant improvement when compared to the results obtained using two exponentials. Therefore, we concluded that the use of two exponential functions is adequate to accurately represent the kinetics embedded in the TRXL data.

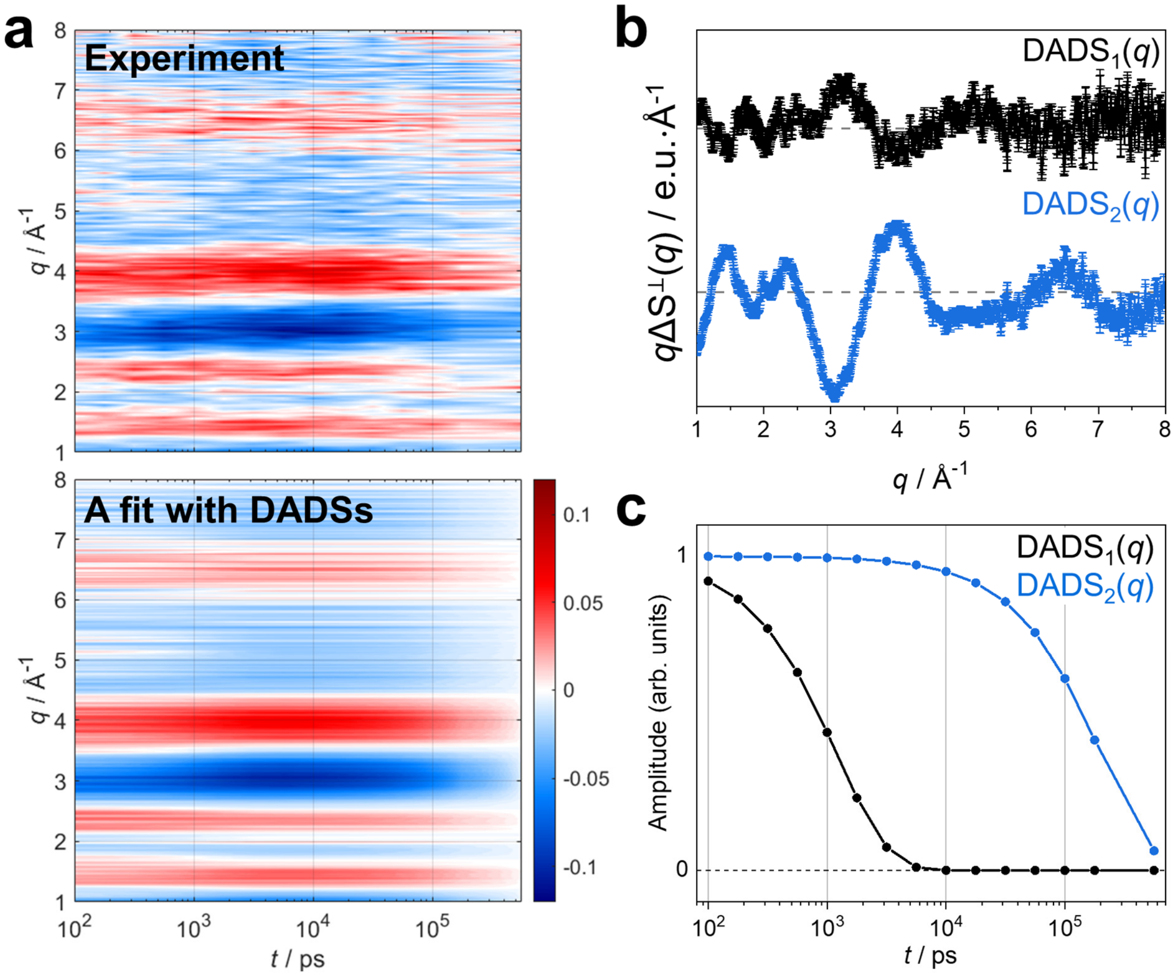

Figure S9. Kinetics-constrained analysis (KCA) of the PEPC-treated experimental data. (a) PEPC-treated experimental data (top) and simulated data (bottom) obtained by KCA. The KCA utilized two exponential time constants, which had been determined from exponential fitting analysis of the RSVs, leading to the extraction of two DADSs along with their respective decay profiles, detailed in the "Kinetic Analysis" section of Supporting Information (b–c). (b) Two DADSs extracted from KCA, $DADS_1$ and $DADS_2$. (c) Time-resolved contributions of each DADS.

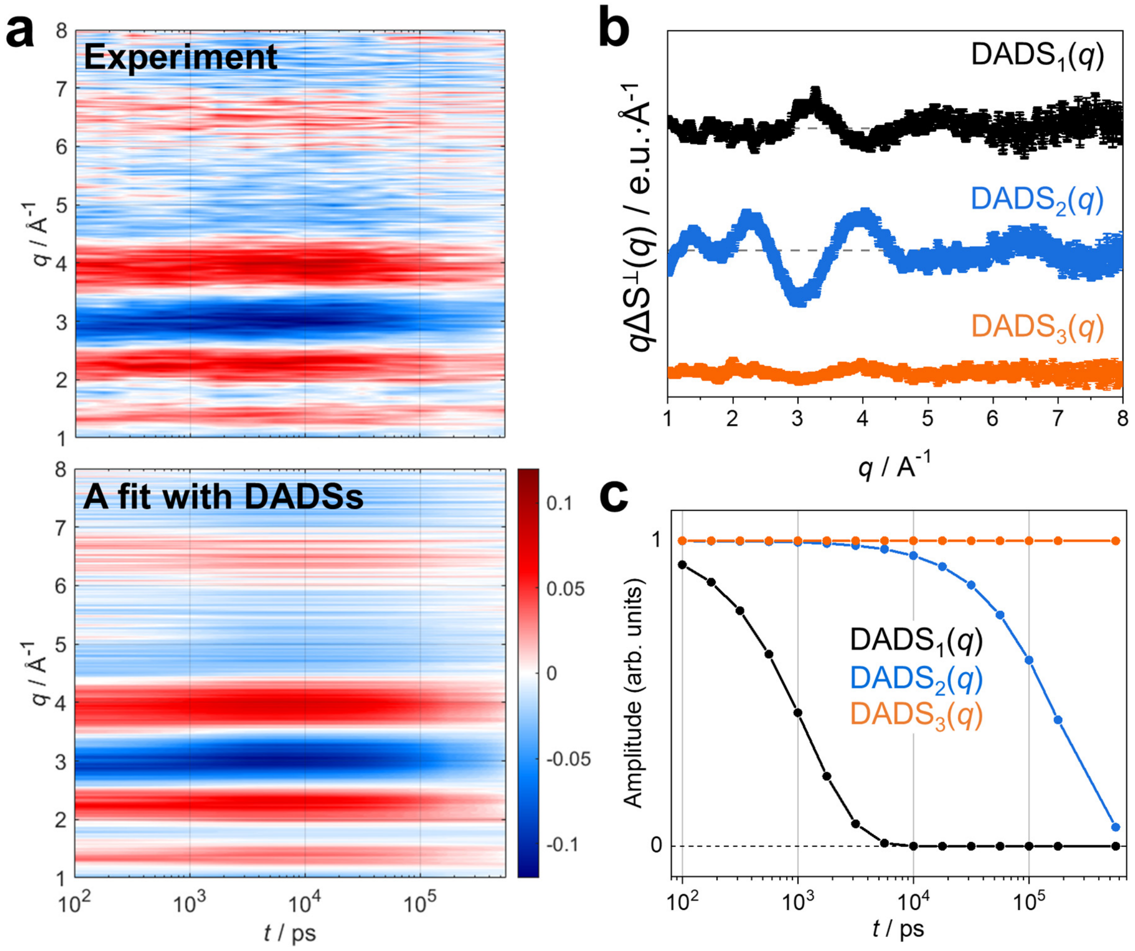

Figure S10. Investigation of the potential existence of a long-lived intermediate or product that does not fully decay to the ground state within the measured time window. To confirm this, we included the third decay-associated difference scattering curve ($DADS_3$), corresponding to a long-lived species, in the analysis of the experimental data. (a) PEPC-treated experimental data (top) and simulated data (bottom) reconstructed using the result of kinetics-constrained analysis (KCA) to extract the DADSs from the PEPC-treated experimental data. (b) Three DADSs extracted from KCA, $DADS_1$, $DADS_2$ and $DADS_3$. (c) Time-resolved contributions of each DADS. $DADS_3(q)$, representing the difference scattering curve of the long-lived product state, shows no significant features in q space, suggesting that the long-lived state is either absent or negligible.

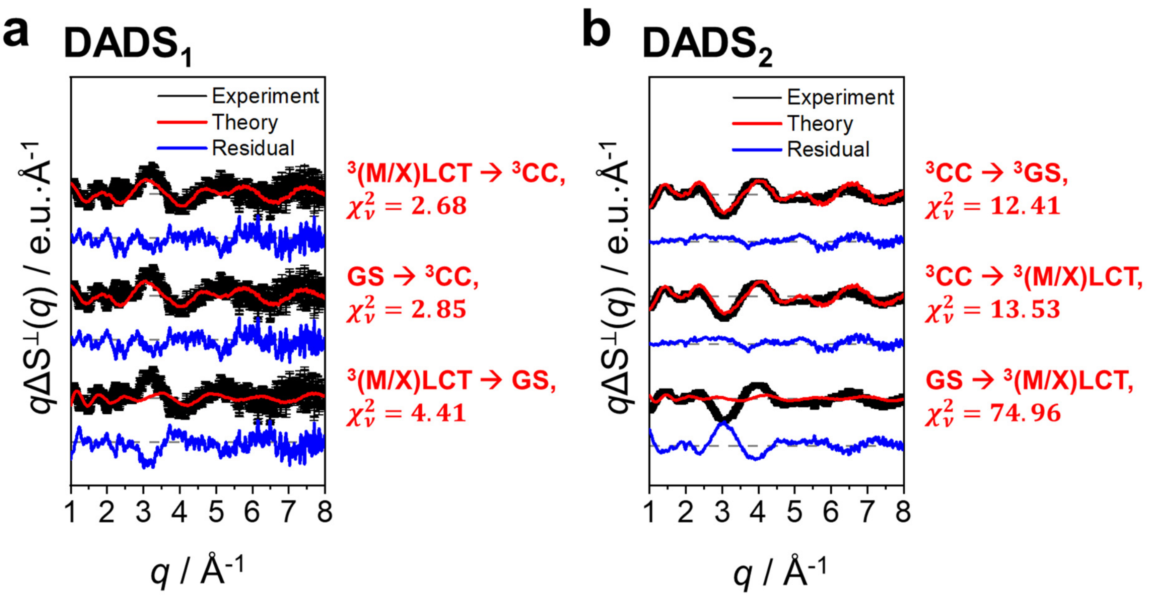

Figure S11. Fit results of DADSs using structures of GS and two excited states obtained by DFT calculations. Panels (a) and (b) showcase experimental $DADS_1(q)$ and $DADS_2(q)$ (black), respectively, alongside their corresponding theoretical fits (red) based on DFT structures. The blue line indicates the residual between the experimental and theoretical DADSs. We note that in this fitting process, the DFT-optimized structures were used as-is, without any adjustment of the structural parameters.

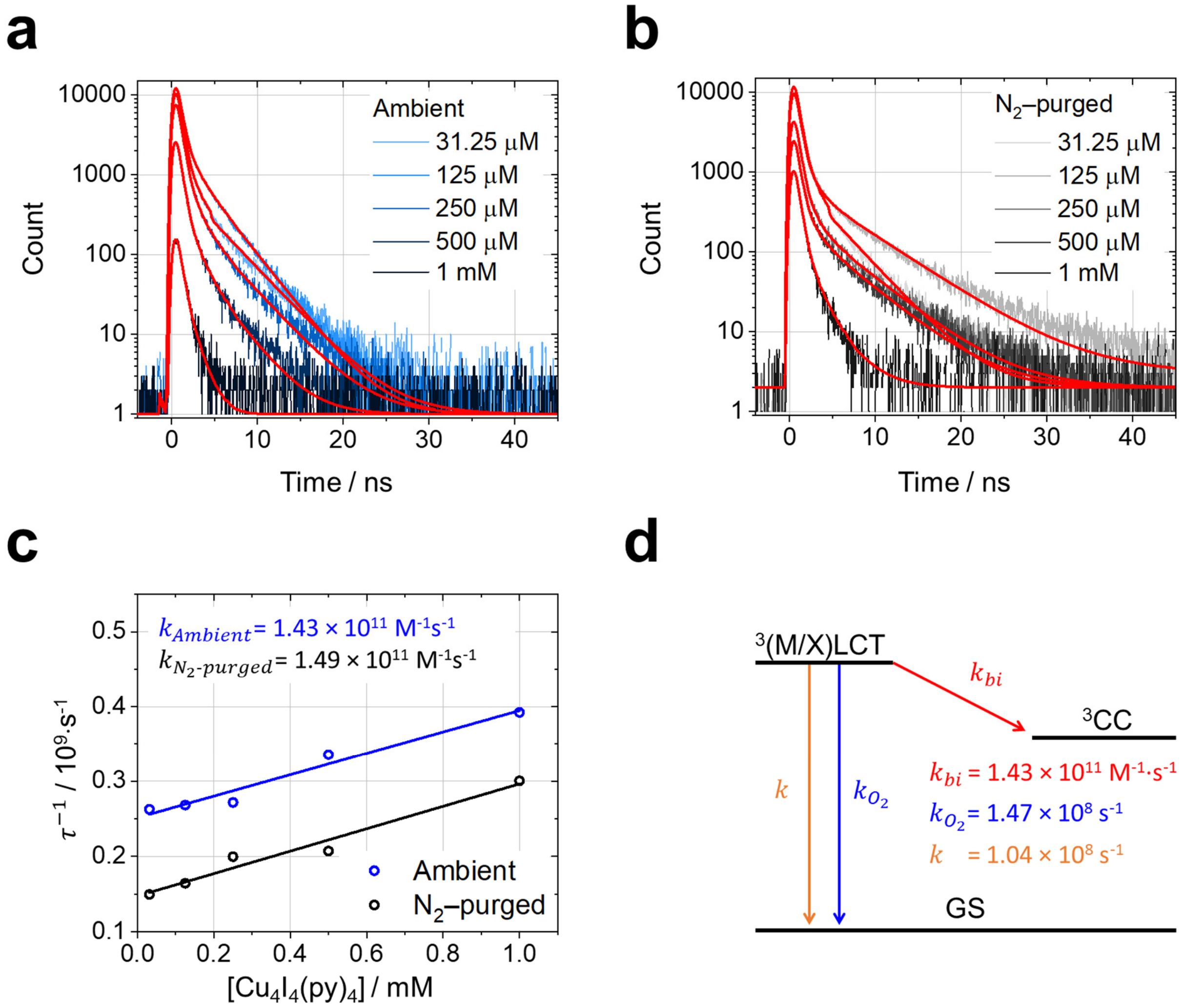

Figure S12. Time-correlated single photon counting (TCSPC) measurements and kinetic model accounting for the observed concentration dependence of the luminescence lifetime. (a–b) TCSPC profiles of $Cu_4I_4(py)_4$ in acetonitrile at concentrations ranging from 31.25 μM to 1 mM, recorded under (a) ambient conditions (light blue to black) and (b) N_2-purged conditions (light gray to black). The samples were excited at 280 nm, and the luminescence lifetime was measured at an emission wavelength of 315 nm. Red lines indicate fits to the experimental data. (c) The plot of τ^{-1} (inverse of the decay lifetime, τ, of the ^{3}(M/X)LCT state) as a function of the concentration of $Cu_4I_4(py)_4$ in acetonitrile solution, derived from TCSPC measurements. The slope represents the rate constant for the self-quenching bimolecular process. (d) Proposed kinetic model explaining the observed concentration-dependent decay lifetime.

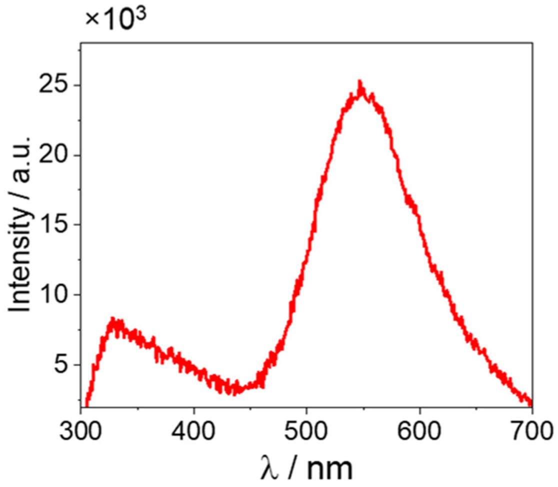

Figure S13. Measured emission spectrum of $Cu_4I_4(py)_4$ in acetonitrile at 293 K. The excitation wavelength is 267 nm. The exposure time of the detector for each data point was set to 1 second, with a slit width of 5 nanometers.

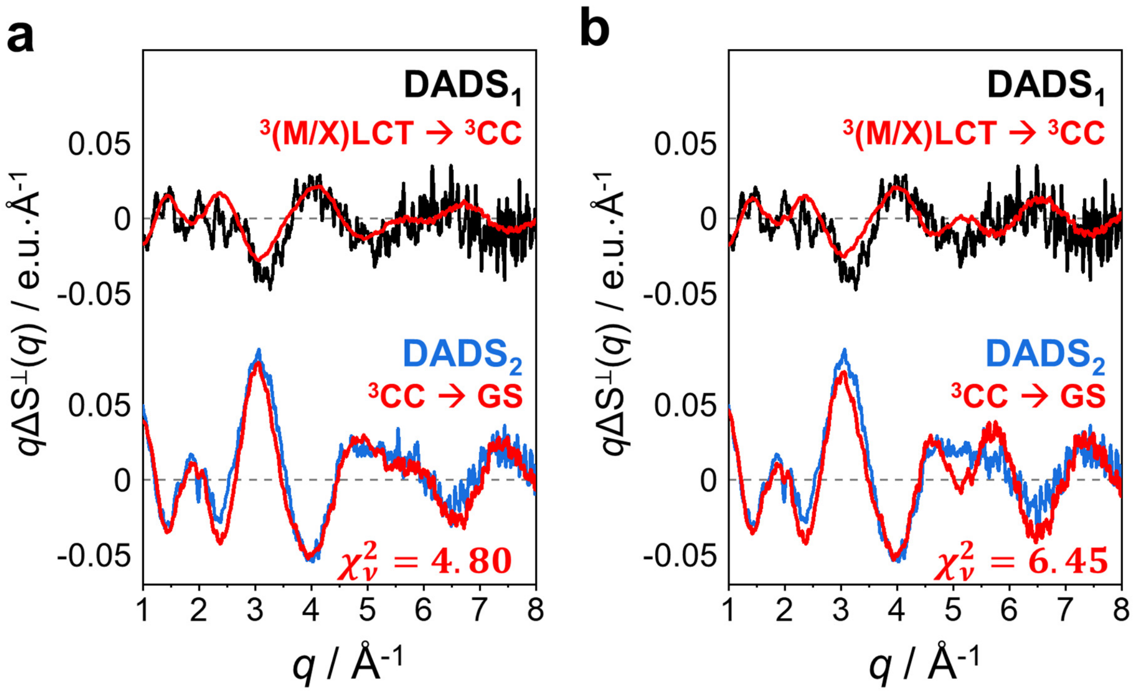

Figure S14. Comparison of the quality of the fit before and after structure refinement. (a) Comparison between the experimental curves and theoretical fits obtained after structure refinement, where the Cu atom positions were fixed and only the I atom positions were optimized; (b) Comparison between the experimental curves and theoretical curves generated directly from the unmodified DFT-optimized structures without further refinement. The experimental $DADS_1$ and $DADS_2$ are shown in black and blue, respectively, with their corresponding theoretical fits displayed in red.

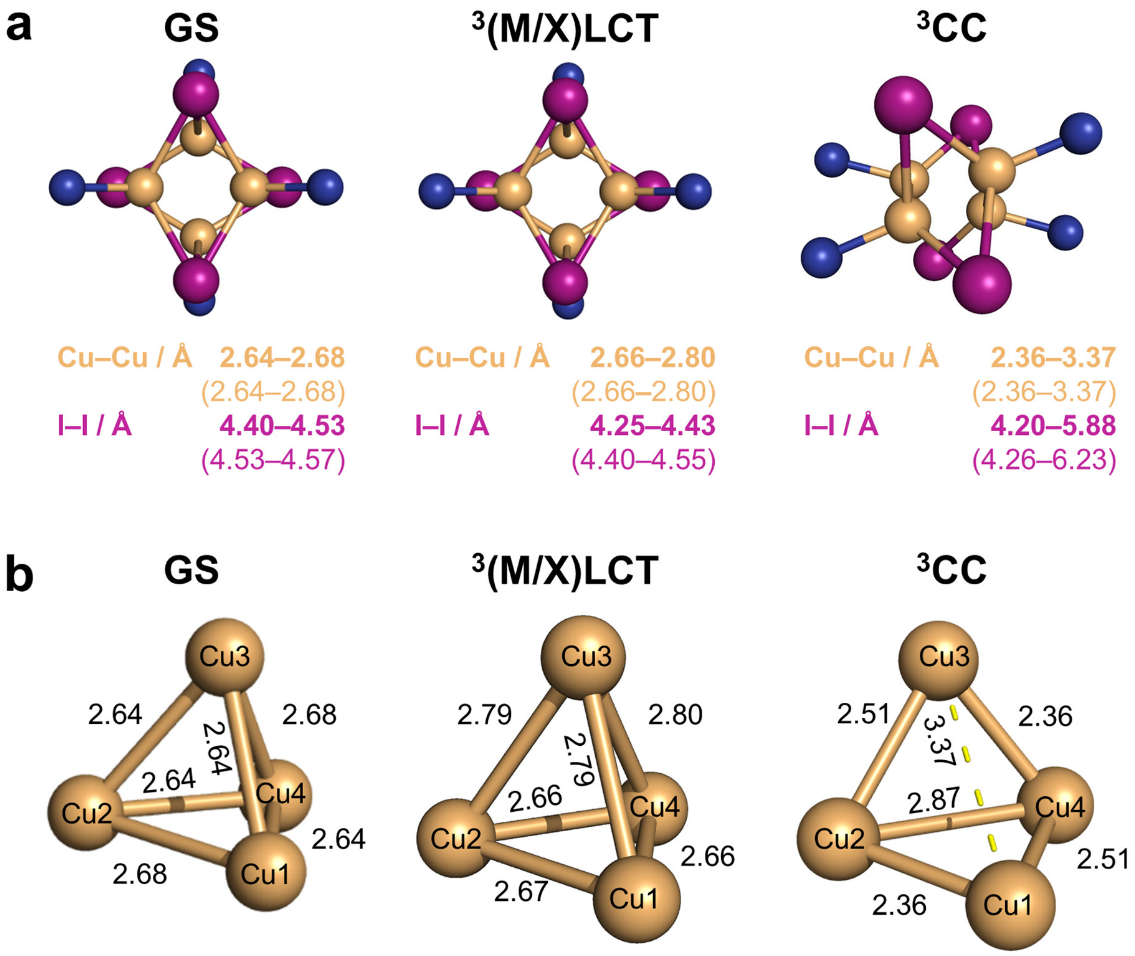

Figure S15. Molecular structures of the GS, ^{3}(M/X)LCT and ^{3}CC states obtained via structure refinement. (a) Top views of the GS (left), ^{3}(M/X)LCT (center) and ^{3}CC (right) structures and the distribution of interatomic distances in the Cu_4I_4 frame. To enhance clarity in the visualization, only the Cu, I, N atoms were depicted in the figure. Here, the distance values obtained through the structure refinement are indicated in bold, and the distance values obtained from DFT calculations are shown in parentheses. (b) Cu_4 cores of the GS (left), ^{3}(M/X)LCT and ^{3}CC structures. The labels of Cu atoms of the Cu_4 core are consistent with those in Figure 4b. In this representation, Solid lines connect atoms that are within 2.98 Å of each other, corresponding to the region where the binding energy of Cu–Cu is reported as −0.175 eV.[36] Distances exceeding this threshold are delineated using dotted lines.

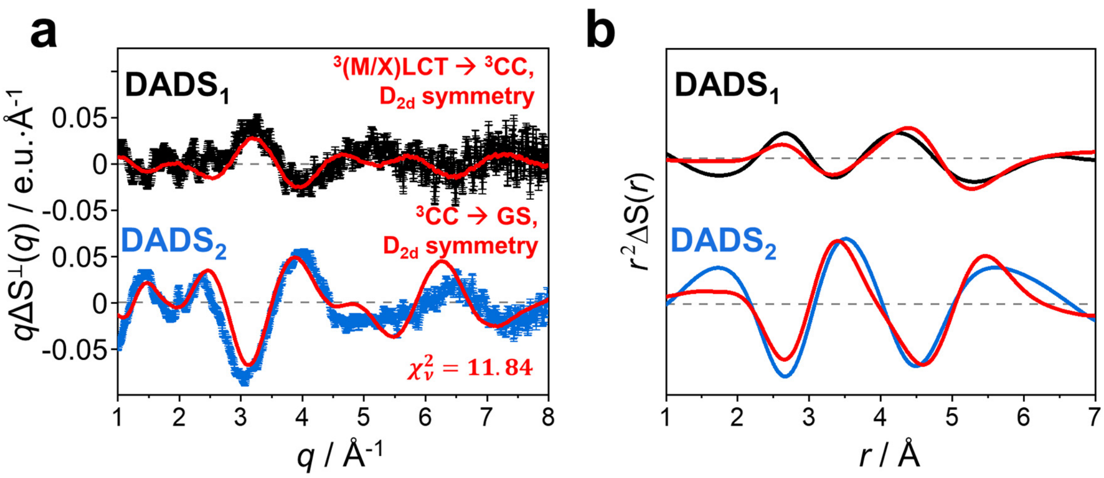

Figure S16. Fit results using D_{2d} symmetry constraints for two excited state structures. (a) Experimental DADSs, DADS(q), and their corresponding theoretical fit, obtained by applying symmetry constraints. (b) Fourier sine transform of the data in (a).

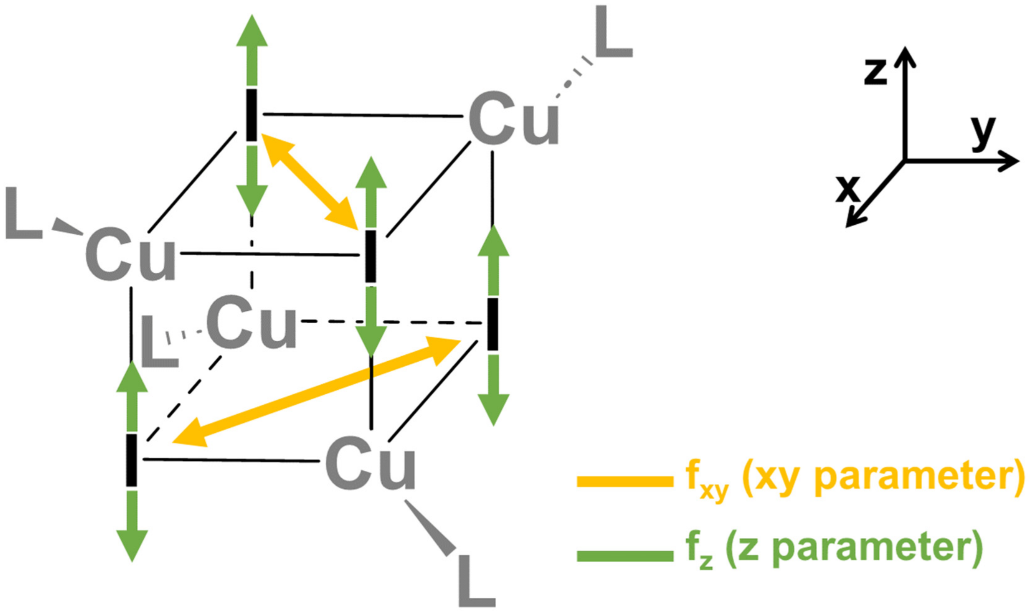

Figure S17. Structure parameters employed in the structure refinement process. The parameters utilized in the structure refinement can be categorized into two categories: those governing modifications to the cluster's atomic positions within the xy-plane, and those affecting positions along the z-direction.The former, xy parameter, denoted as f_{xy}, quantifies the overall expansion or contraction of the molecular structure within the xy-plane. The other parameter, denoted as f_z, manages the expansion or contraction along the z-axis. Accordingly, f_{xy} primarily governs the distances between each X–X pair (where X = I) within the same xy-plane, enabling elongation or contraction. Conversely, f_z predominantly contributes to structural alterations along the z-axis. The ligands denoted by the symbol "L" refer to the pyridine molecules connected to adjacent copper atoms. The Cu atoms and their ligands, denoted as "L", are depicted in gray to indicate that their positions are fixed during the structural fitting.

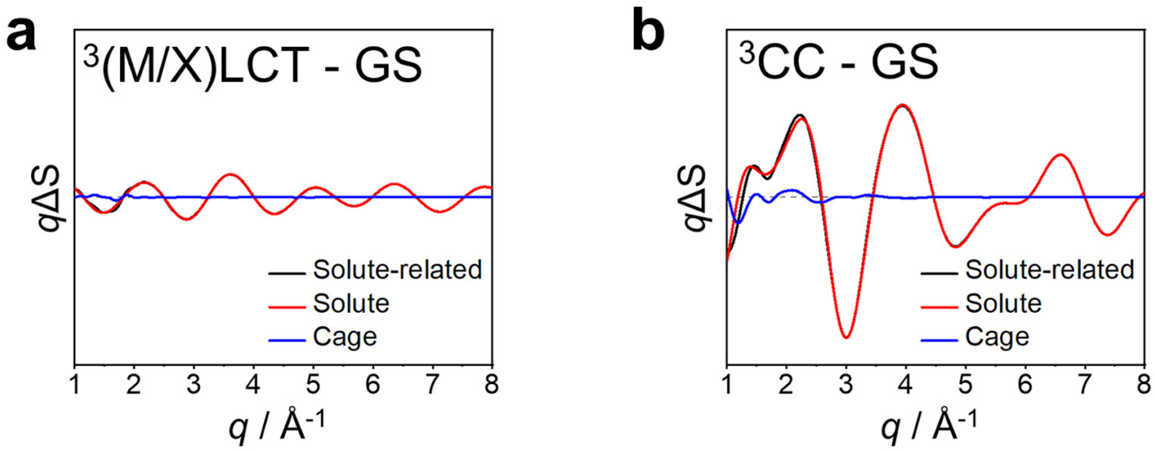

Figure S18. Solute-related terms of the difference scattering signals, along with their constituent solute and cage terms, for ^{3}(M/X)LCT and ^{3}CC. The cage term is derived from MD simulation snapshots. (a–b) Difference scattering curves for the solute-related terms (black), solute terms (red), and cage terms (blue) for (a) the ^{3}(M/X)LCT state and (b) the ^{3}CC state.

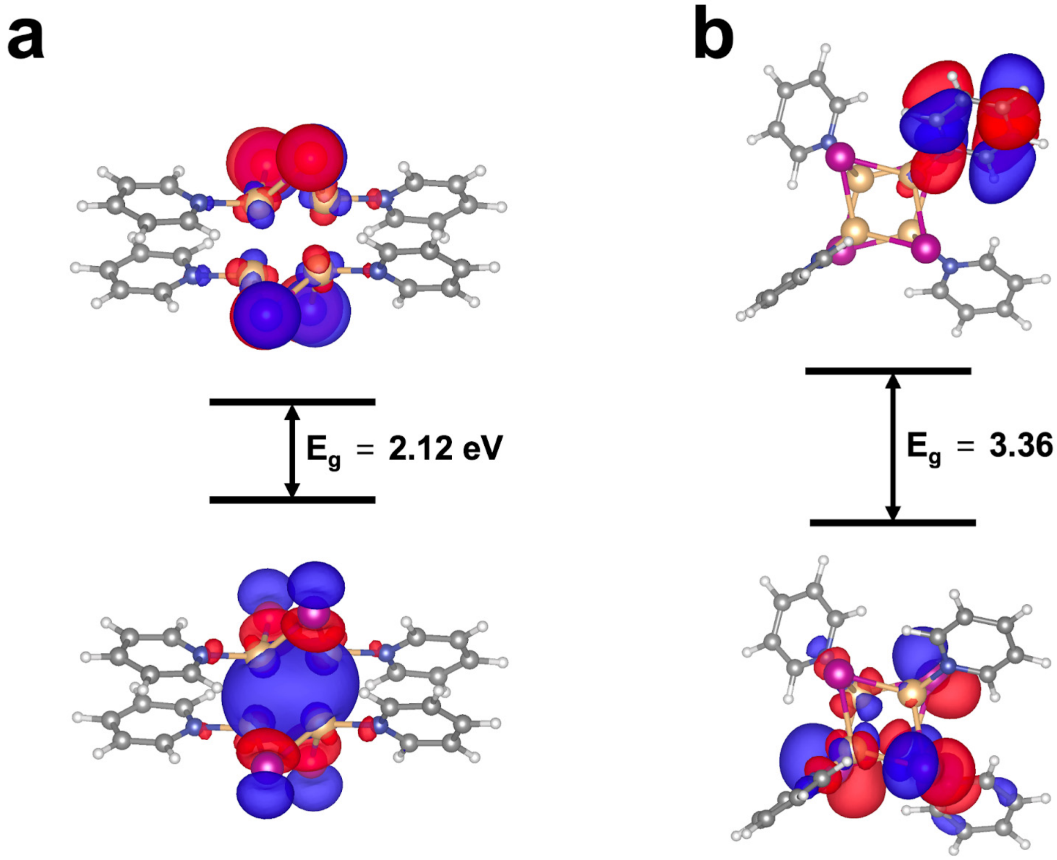

Figure S19. Lowest singly occupied molecular orbital (LSOMO) and highest singly occupied molecular orbital (HSOMO) of two triplet structures. (a) LSOMO and HSOMO of the ^{3}CC structure and (b) LSOMO and HSOMO of the ^{3}(M/X)LCT structure. Note that the LSOMO and HSOMO of the ^{3}CC and ^{3}(M/X)LCT structures exhibit distinctly different features.

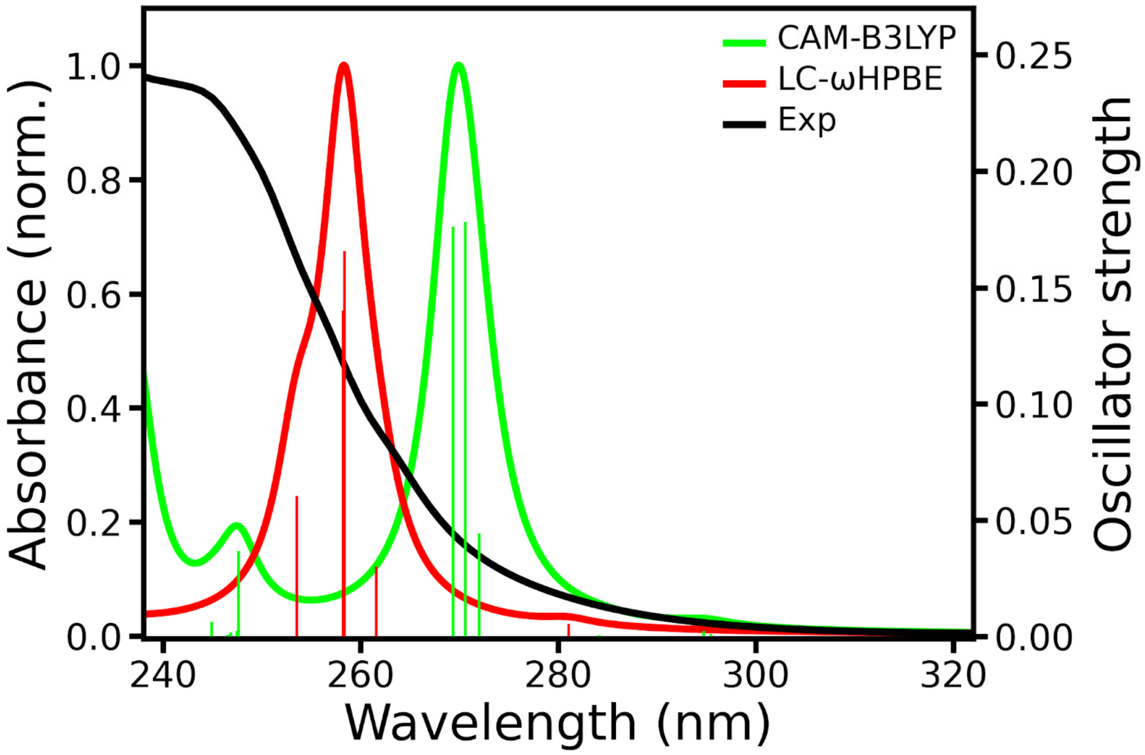

Figure S20. Calculated absorption spectrum and oscillator strengths. Here, we compared the experimental and calculated absorption spectra. Taking into account the typical error in excitation energy associated with TD-DFT calculations, the calculated spectrum effectively reproduces the first peak observed in the experimental spectrum. The height of the vertical lines represents the corresponding oscillator strength values of the calculated transitions. All spectra are normalized, and a 0.1 eV Lorentzian broadening was applied to the calculated spectra.

Reference

[1] E. H. Choi, D.-S. Ahn, S. Park, C. Kim, C. W. Ahn, S. Kim, M. Choi, C. Yang, T. W. Kim, H. Ki, J. Choi, M. N. Pedersen, M. Wulff, J. Kim, H. Ihee, *J. Phys. Chem. Lett.* **2019**, *10*, 1279.

[2] Y. Lee, H. Ki, D. Im, S. Eom, J. Gu, S. Lee, J. Kim, Y. Cha, K. W. Lee, S. Zerdane, M. Levantino, H. Ihee, *J. Am. Chem. Soc.* **2023**, *145*, 23715.

[3] C. W. Ahn, H. Ki, J. Kim, J. Kim, S. Park, Y. Lee, K. H. Kim, Q. Kong, J. Moon, M. N. Pedersen, M. Wulff, H. Ihee, *J. Phys. Chem. Lett.* **2018**, *9*, 647.

[4] T. K. Kim, J. H. Lee, M. Wulff, Q. Kong, H. Ihee, *ChemPhysChem* **2009**, *10*, 1958.

[5] H. Ihee, M. Wulff, J. Kim, S.-i. Adachi, *Int. Rev. Phys. Chem.* **2010**, *29*, 453.

[6] M. Cammarata, M. Lorenc, T. Kim, J.-H. Lee, Q. Kong, E. Pontecorvo, M. Lo Russo, G. Schiro, A. Cupane, M. Wulff, *J. Chem. Phys.* **2006**, *124*, 124504.

[7] K. S. Kjær, T. B. Van Driel, J. Kehres, K. Haldrup, D. Khakhulin, K. Bechgaard, M. Cammarata, M. Wulff, T. J. Sørensen, M. M. Nielsen, *Phys. Chem. Chem. Phys.* **2013**, *15*, 15003.

[8] H. Ki, J. Gu, Y. Cha, K. W. Lee, H. Ihee, *Struct. Dyn.* **2023**, *10*, 034103.

[9] H. Ki, T. W. Kim, J. Moon, J. Kim, Y. Lee, J. Heo, K. H. Kim, Q. Kong, D. Khakhulin, G. Newby, *Chem. Commun.* **2022**, *58*, 7380.

[10] J. E. Loefroth, *J. Phys. Chem.* **1986**, *90*, 1160.

[11] F. James, M. Roos, *Comput. Phys. Commun.* **1975**, *10*, 343.

[12] J. Heo, D. Kim, A. Segalina, H. Ki, D.-S. Ahn, S. Lee, J. Kim, Y. Cha, K. W. Lee, J. Yang, J. P. F. Nunes, X. Wang, H. Ihee, *Nature* **2024**, *625*, 710.

[13] K. Refson, *Comput. Phys. Commun.* **2000**, *126*, 310.

[14] S. Nosé, *Mol. Phys.* **1984**, *52*, 255.

[15] W. G. Hoover, *Phys. Rev. A* **1985**, *31*, 1695.

[16] A. K. Rappe, C. J. Casewit, K. S. Colwell, W. A. Goddard, III, W. M. Skiff, *J. Am. Chem. Soc.* **1992**, *114*, 10024.

[17] C. Adamo, V. Barone, *J. Chem. Phys.* **1999**, *110*, 6158.

[18] A. D. Becke, *J. Chem. Phys.* **1993**, *98*, 5648.

[19] J.-D. Chai, M. Head-Gordon, *J. Chem. Phys.* **2008**, *128*, 084106.

[20] T. Yanai, D. P. Tew, N. C. Handy, *Chem. Phys. Lett.* **2004**, *393*, 51.

[21] S. Grimme, J. Antony, S. Ehrlich, H. Krieg, *J. Chem. Phys.* **2010**, *132*, 154104.

[22] S. Mai, F. Plasser, J. Dorn, M. Fumanal, C. Daniel, L. González, *Coord. Chem. Rev.* **2018**, *361*, 74.

[23] F. Weigend, R. Ahlrichs, *Phys. Chem. Chem. Phys.* **2005**, *7*, 3297.

[24] V. Barone, M. Cossi, *J. Phys. Chem. A* **1998**, *102*, 1995.

[25] M. J. Frisch, G. W. Trucks, H. B. Schlegel, G. E. Scuseria, M. A. Robb, J. R. Cheeseman, G. Scalmani, V. Barone, G. A. Petersson, H. Nakatsuji, X. Li, M. Caricato, A. V. Marenich, J. Bloino, B. G. Janesko, R. Gomperts, B. Mennucci, H. P. Hratchian, J. V. Ortiz, A. F. Izmaylov, J. L. Sonnenberg, Williams, F. Ding, F. Lipparini, F. Egidi, J. Goings, B. Peng, A. Petrone, T. Henderson, D. Ranasinghe, V. G. Zakrzewski, J. Gao, N. Rega, G. Zheng, W. Liang, M. Hada, M. Ehara, K. Toyota, R. Fukuda, J. Hasegawa, M. Ishida, T. Nakajima, Y. Honda, O. Kitao, H. Nakai, T. Vreven, K. Throssell, J. A. Montgomery Jr., J. E. Peralta, F. Ogliaro, M. J. Bearpark, J. J. Heyd, E. N. Brothers, K. N. Kudin, V. N. Staroverov, T. A. Keith, R. Kobayashi, J. Normand, K. Raghavachari, A. P. Rendell, J. C. Burant, S. S. Iyengar, J. Tomasi, M. Cossi, J. M. Millam, M. Klene, C. Adamo, R. Cammi, J. W. Ochterski, R. L. Martin, K. Morokuma, O. Farkas, J. B. Foresman, D. J. Fox,*Gaussian 16 Rev. C.01*, Gaussian, Inc., Wallingford, CT, **2016**.

[26] F. Plasser, *J. Chem. Phys.* **2020**, *152*, 084108.

[27] K. R. Kyle, C. K. Ryu, P. C. Ford, J. A. DiBenedetto, *J. Am. Chem. Soc.* **1991**, *113*, 2954.

[28] P. C. Ford, E. Cariati, J. Bourassa, *Chem. Rev.* **1999**, *99*, 3625.

[29] A. Vogler, H. Kunkely, *J. Am. Chem. Soc.* **1986**, *108*, 7211.

[30] P. C. Ford, A. Vogler, *Acc. Chem. Res.* **1993**, *26*, 220.

[31] M. Vitale, W. E. Palke, P. C. Ford, *J. Phys. Chem.* **1992**, *96*, 8329.

[32] F. De Angelis, S. Fantacci, A. Sgamellotti, E. Cariati, R. Ugo, P. C. Ford, *Inorg. Chem.* **2006**, *45*, 10576.

[33] K. R. Kyle, P. C. Ford, *J. Am. Chem. Soc.* **1989**, *111*, 5005.

[34] K. R. Kyle, J. DiBenedetto, P. C. Ford, *J. Chem. Soc., Chem. Commun.* **1989**, *11*, 714.

[35] M. Vitale, C. K. Ryu, W. E. Palke, P. C. Ford, *Inorg. Chem.* **1994**, *33*, 561.

[36] P. K. Mehrotra, R. Hoffmann, *Inorg. Chem.* **1978**, *17*, 2187.
